# Supplementary figures and images for: USP39 promotes antiviral defense through post-transcriptional control of RIG-I and stabilization of STING
Source: PLoS Biol. 2026 May 11;24(5):e3003796. doi: 10.1371/journal.pbio.3003796 (PMC13178990; doi:10.1371/journal.pbio.3003796)

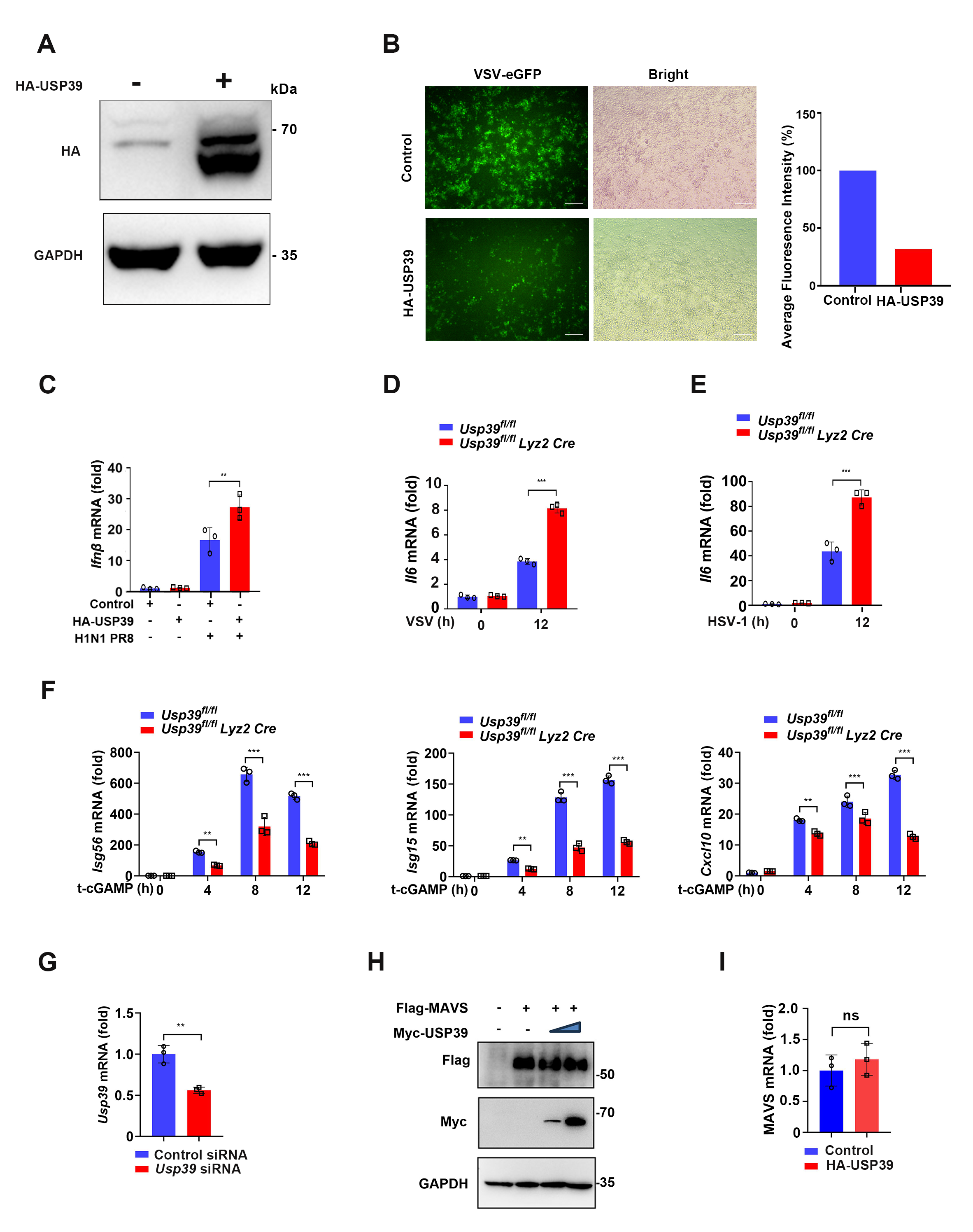

Supplement: S1 Fig — (A) The efficiency of HA-USP39 overexpression in HEK293T cells. (B) HeLa cells overexpressed a control vector or HA-USP39, and then were infected with VSV-eGFP (MOI = 1) for 12 h before examination by fluorescence microscopy. Scale bar = 100 μm. (C) HeLa cells overexpressed a control vector or HA-USP39, and then were infected with H1N1 PR8 (MOI = 1) for 12 h before Ifn-β mRNA levels were measured by qPCR. (D, E) Usp39fl/fl and Usp39fl/fl Lyz2 Cre macrophages were infected with VSV (MOI = 1) (D) or HSV-1 (MOI = 10) (E) for the indicated time before Il-6 mRNA levels were measured by qPCR. (F) Usp39fl/fl and Usp39fl/fl Lyz2 Cre macrophages were transfected with cGAMP (1 μg/mL) for the indicated time, before Isg15, Isg56, and Cxcl10 mRNA levels were measured by qPCR. (G) Usp39 was knocked down in macrophages, and Usp39 mRNA levels were measured by qPCR. (H, I) Flag-MAVS and Myc-USP39 (H) or Myc-USP39 (I) were overexpressed in HEK293T cells. The cell lysates were analyzed by western blotting, and MAVS mRNA was detected by qPCR. The data represent the means ± SD, from three independent experiments. *p < 0.05, **p < 0.01, ***p < 0.001 using Student t test. This data underlying this Figure can be found in S1 Data and S1 Raw Images. (TIF) [file pbio.3003796.s001.tif]

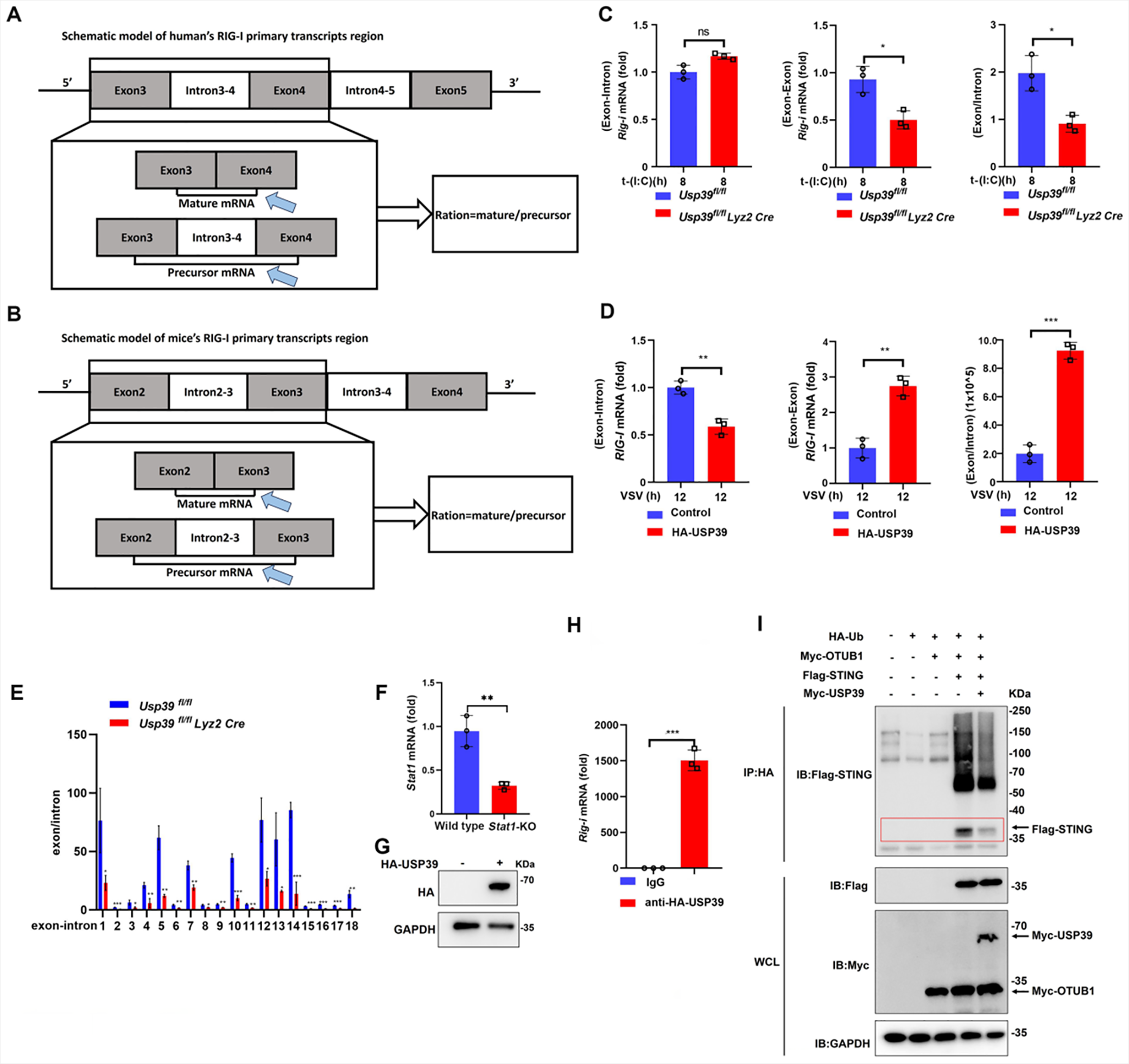

Supplement: S2 Fig — (A, B) The region from exon 3 to 4 in the human Rig-i primary transcript (A) and exon 2 to 3 in the mouse Rig-i primary transcript (B) was used to design specific primers to detect spliced and unspliced Rig-i mRNA. (C, D) Special Rig-i mRNA levels were measured by qPCR in Usp39fl/fl and Usp39fl/fl Lyz2 Cre macrophages after they had been transfected with Poly (I:C) (1 μg/mL) at the indicated time (C). A control vector or HA-USP39 were overexpressed in HeLa cells before being infected with VSV (MOI = 1) at the indicated time. Special Rig-i mRNA levels were measured by qPCR (D). (E) All the 18 exons/introns in BMDM were detected by qPCR. (F) Stat1 mRNA in Stat1 Ko-L929 cell were detected by qPCR. (G) HA-USP39 overexpressed in STAT1 Ko-L929 cell, and the cell lysates were analyzed by western blotting. (H) Myc-RIG-I and HA-USP39 were co-overexpressed in HEK293T cells and performed by RIP, Rig-i mRNA levels measured by qPCR. (I) Flag-STING, HA-Ub, Myc-OTUB1, Myc-USP39 were co-overexpressed in HEK293T cells before the cells were treated with MG132 (20 μM) for 6 h. The cell lysates were precipitated with anti-HA magnetic beads, and Flag-STING protein was determined by western blotting. The data represent the means ± SD, from three independent experiments. *p < 0.05, **p < 0.01, ***p < 0.001 using Student t test. This data underlying this Figure can be found in S1 Data and S1 Raw Images. (TIF) [file pbio.3003796.s002.tif]

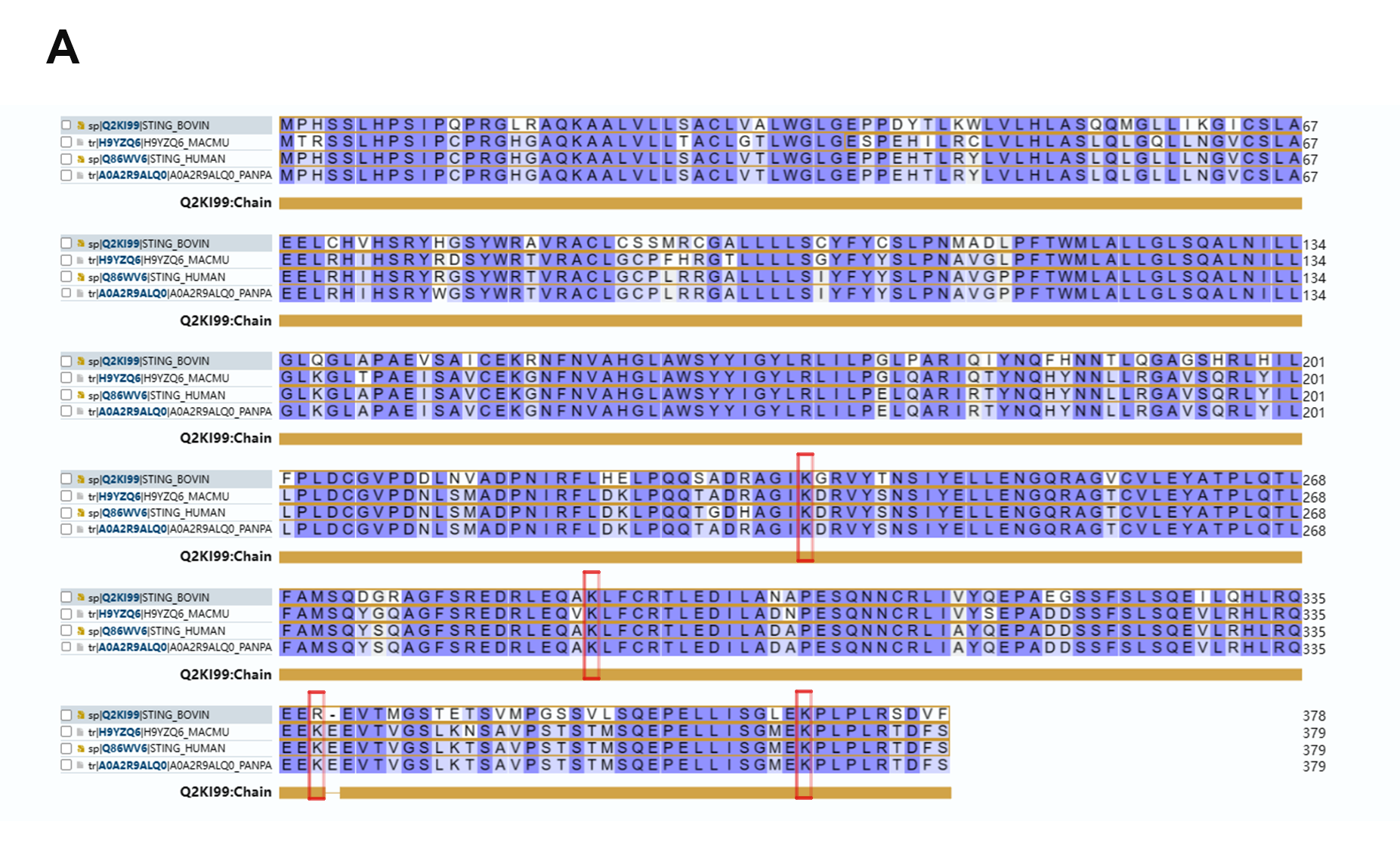

Supplement: S3 Fig — (A) Conserved lysine sites in different species. (TIF) [file pbio.3003796.s003.tif]
