# Supplementary material for: USP39 promotes antiviral defense through post-transcriptional control of RIG-I and stabilization of STING
Source: PLoS Biol. 2026 May 11;24(5):e3003796. doi: 10.1371/journal.pbio.3003796 (PMC13178990; doi:10.1371/journal.pbio.3003796)
Supplement: S1 Table — (DOCX) [file pbio.3003796.s004.docx]

| **S1 Table.** Primers used for this study | | |
| --- | --- | --- |
| Gene name |  | Primer sequence, 5’-3’ |
| HSV-2 ICP27 | Forward | TGTCGGAGATCGACTACACG |
|  | Reverse | GGTGCGTGTCCAGTATTTCA |
| VSV-N | Forward | CCTTGCAGTGACATGACTGCTCTT |
|  | Reverse | TGATAGTACCGGAGGATTGACGAC |
| HSV-1 UL30 | Forward | CATCACCGACCCGGAGAGGGAC |
|  | Reverse | GGGCCAGGCGCTTGTTGGTGTA |
| H1N1 PR8 | Forward | TTCTAACCGAGGTCGAAACG |
|  | Reverse | ACAAAGCGTCTACGCTGCAG |
| Mouse *Sting* | Forward | AGTCCTGCTAGGTGTCCACT |
|  | Reverse | CTTCTGAATGGGGAGACAGCA |
| Mouse *Usp39* | Forward | GAGGTGCGAGCAAAGAATGG |
|  | Reverse | CAGGCGTATGCGTTGATGTG |
| Mouse *Isg56* | Forward | GCAGAGAGTCAAGGCAGGTT |
|  | Reverse | GCATTCTCTCCCATGGTTGC |
| Mouse *Isg15* | Forward | AGCAATGGCCTGGGACCTAA |
|  | Reverse | GGAGTTAGTCACGGACACCA |
| Mouse *Cxcl10* | Forward | CCACGTGTTGAGATCATTGCC |
|  | Reverse | GAGGCTCTCTGCTGTCCATC |
| Mouse intron *Rig-i* | Forward | TCCCCCAGATCCGAGACAC |
|  | Reverse | TTGCTGTTGTCTTTCTCC |
| Mouse exon *Rig-i* | Forward | AGCCATCGAAAGTTGGGACT |
|  | Reverse | AGTGTCTCGGATCTGTCTGA |
| Human intron *Rig-i* | Forward | GTCCACCTTCAGGTACCTG |
|  | Reverse | AGGAGAGAATGGCATTCTA |
| Human exon *Rig-i* | Forward | CCACTGCCTCAGGTCGT |
|  | Reverse | GTTCCTCGGAGACATCCTTG |
| Human *Rig-i* | Forward | TGATCCAAACCAGAGGCAGAG |
|  | Reverse | GTCCCATGTCTGAAGGCGTAA |
| Mouse *Rig-I* | Forward | AGCCATCGAAAGTTGGGACT |
|  | Reverse | AGTGTCTCGGATCTGTCTGA |
| Mouse *Il6* | Forward | TAGTCCTTCCTACCCCAATTTCC |
|  | Reverse | TTGGTCCTTAGCCACTCCTTC |
| Mouse *Ifn-b* | Forward | ATGAGTGGTGGTTGCAGGC |
|  | Reverse | TGACCTTTCAAATGCAGTAGA TTCA |
| Mouse *Ifn-a4* | Forward | ACCCACAGCCCAGAGAGTGACC |
|  | Reverse | AGGCCCTCTTGTTCCCGAGGT |
| Mouse *b-actin* | Forward | AGTGTGACGTTGACATCCGT |
|  | Reverse | GCAGCTCAGTAACAGTCCGC |
| Human *Ifn-b* | Forward | CATTACCTGAAGGCCAAGGA |
|  | Reverse | CAATTGTCCAGTCCCAGAGG |
| Human *b-actin* | Forward | TGGAGAAAATCTGGCACCACACC |
|  | Reverse | GATGGGCACAGTGTGGGTGACCC |
| Mouse Mavs | Forward | CTTCATCTGCGAGGGCTCTG |
| Mouse Mavs | Reverse | TGGACTGAGATGGACAAGAGA |
| Mouse *Stat1* | Forward | AGCTCTGCTCCATACCCTGA |
| Mouse *Stat1* | Reverse | TTCCCTCCTGGGCCTGATTA |
| Mouse - Exon - Intron 1 | Forward | CAGCGCCCTGAGGCAGTTTCG |
| Mouse - Exon 1 | Reverse | GATATAGTCTCTGAATGCTTG |
| Mouse - Exon - Intron 2 | Forward | TGTTTGCAGAGGAGGTGCAG |
| Mouse - Exon 2 | Reverse | CTGCATGGTACAGGGC |
| Mouse - Exon - Intron 3 | Forward | TTTTTCAGGTTACTGTGGAC |
| Mouse - Exon 3 | Reverse | TTCAGAAAGGATATCATTT |
| Mouse - Exon - Intron 4 | Forward | TCTTGGTCAGGGATATAAGG |
| Mouse - Exon 4 | Reverse | CTGCCATTCTCCCTTTAGTGT |
| Mouse - Exon - Intron 5 | Forward | ATCACAAAGGTTTCAAAAGGG |
| Mouse - Exon 5 | Reverse | GATGAGAGCAAACGATGCTTGG |
| Mouse - Exon - Intron 6 | Forward | ATATTACAGAAGCGTCTTCTA |
| Mouse - Exon 6 | Reverse | TCCCTTTCTTGGCAGGCAGGGC |
| Mouse - Exon - Intron 7 | Forward | TACTAAAAGGTTGTGGAAA |
| Mouse - Exon 7 | Reverse | AGTTGCCTGCTGCTCATAG |
| Mouse - Exon - Intron 8 | Forward | CATTCCCCAGGTACAACATTGC |
| Mouse - Exon 8 | Reverse | GTGTTATGACACTCATCAAATA |
| Mouse - Exon - Intron 9 | Forward | CTCCTTACAGGTCGTTGGGCTG |
| Mouse - Exon 9 | Reverse | TGCAACGTTGTCTCTGACTGTG |
| Mouse - Exon - Intron 10 | Forward | CTTGGAGCCTGTGGCTTTAAAG |
| Mouse - Exon 10 | Reverse | AGTTCCTCGGAGACATCCTT |
| Mouse - Exon - Intron 11 | Forward | ACCCTGTAGGAAAGCTTTTTCAAA |
| Mouse - Exon 11 | Reverse | TGTGTACAGGAAGAGCGCTTT |
| Mouse - Exon - Intron 12 | Forward | TTCAAACAGAAATACAACG |
| Mouse - Exon 12 | Reverse | TAAGCTCTCGCTCGGTCTCA |
| Mouse - Exon - Intron 13 | Forward | ATTCCTATCTGTGGATTGCTGTAT |
| Mouse - Exon 13 | Reverse | TTTAGAACAAACAGTGGCCTT |
| Mouse - Exon - Intron 14 | Forward | CACTTTTAGGCTCTGAAGAA |
| Mouse - Exon 14 | Reverse | CTGTTGCCCGGTTTGTTC |
| Mouse - Exon - Intron 15 | Forward | TATCCAACACAGGAATGACGCT |
| Mouse - Exon 15 | Reverse | AGAGAATGACGAGATTGCACT |
| Mouse - Exon - Intron 16 | Forward | CTGCAGGCCGAGGAAG |
| Mouse - Exon 16 | Reverse | TTCCTTGATCATGTTCG |
| Mouse - Exon - Intron 17 | Forward | TTCGCATGTAGGTTCACCGCA |
| Mouse - Exon 17 | Reverse | GTCAGCTGTGTAGCACGCA |
| Mouse - Exon - Intron 18 | Forward | CTCCTAGACGTCCCA |
| Mouse - Exon 18 | Reverse | TTGTCATAGATCTTTGG |
