# Supplementary material for: USP39 promotes antiviral defense through post-transcriptional control of RIG-I and stabilization of STING
Source: PLoS Biol. 2026 May 11;24(5):e3003796. doi: 10.1371/journal.pbio.3003796 (PMC13178990; doi:10.1371/journal.pbio.3003796)

Figure 3

I

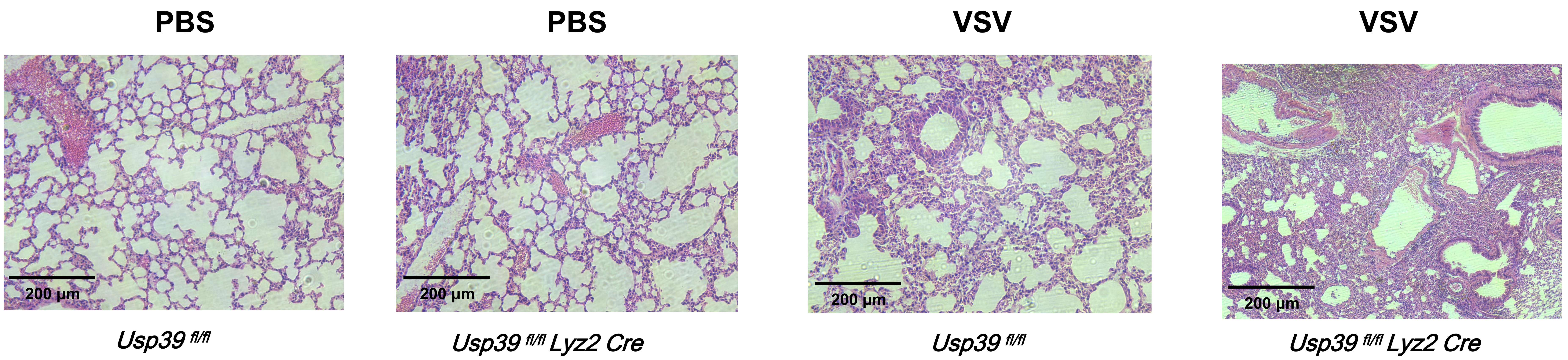
PBS
PBS
VSV
VSV
200 µm
200 µm
200 µm
200 µm
Usp39 fl/fl
Usp39 fl/fl Lyz2 Cre
Usp39 fl/fl
Usp39 fl/fl Lyz2 Cre

R

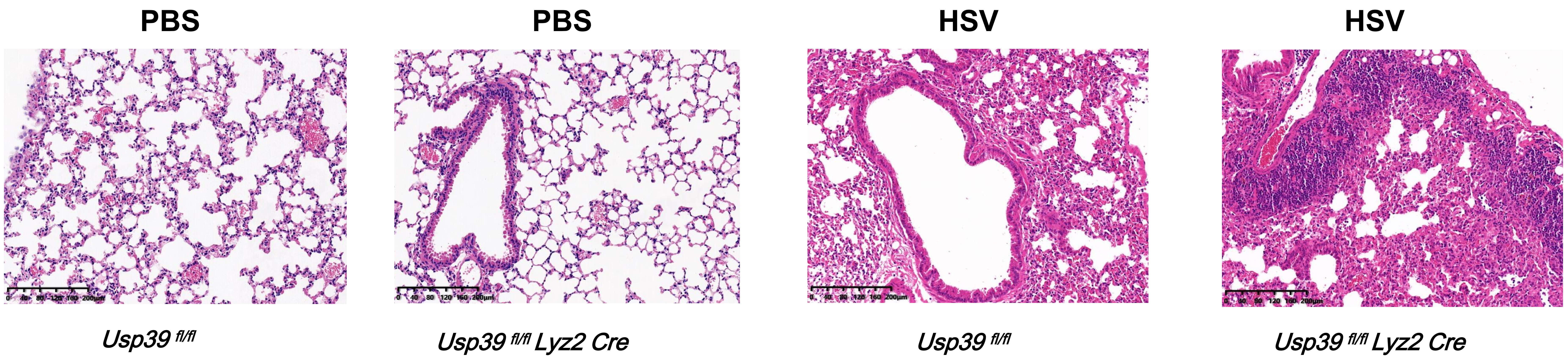
PBS
PBS
HSV
HSV
Usp39 fl/fl
Usp39 fl/fl Lyz2 Cre
Usp39 fl/fl
Usp39 fl/fl Lyz2 Cre

Figure 4

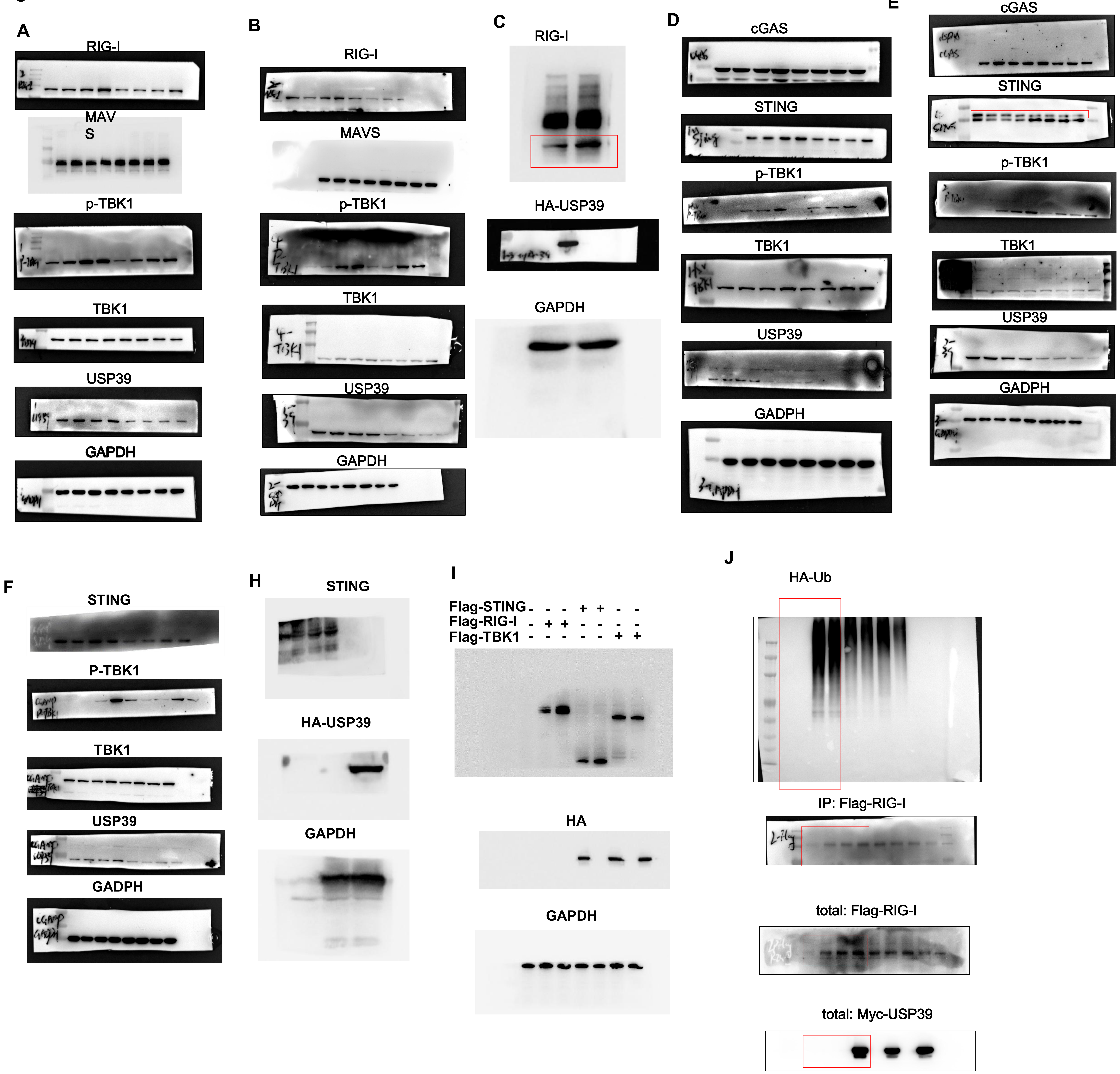
A
RIG-I
MAV
S
p-TBK1
TBK1
USP39
GAPDH
B
RIG-I
MAVS
p-TBK1
TBK1
USP39
GAPDH
C
RIG-I
HA-USP39
GAPDH
D
cGAS
STING
p-TBK1
TBK1
USP39
GADPH
E
cGAS
STING
p-TBK1
TBK1
USP39
GADPH
F
STING
P-TBK1
TBK1
USP39
GADPH
H
STING
HA-USP39
GAPDH
I
Flag-STING - - + + - -
Flag-RIG-I - + + - - - -
Flag-TBK1 - - - - - + +
HA
GAPDH
J
HA-Ub
IP: Flag-RIG-I
total: Flag-RIG-I
total: Myc-USP39

Figure 5

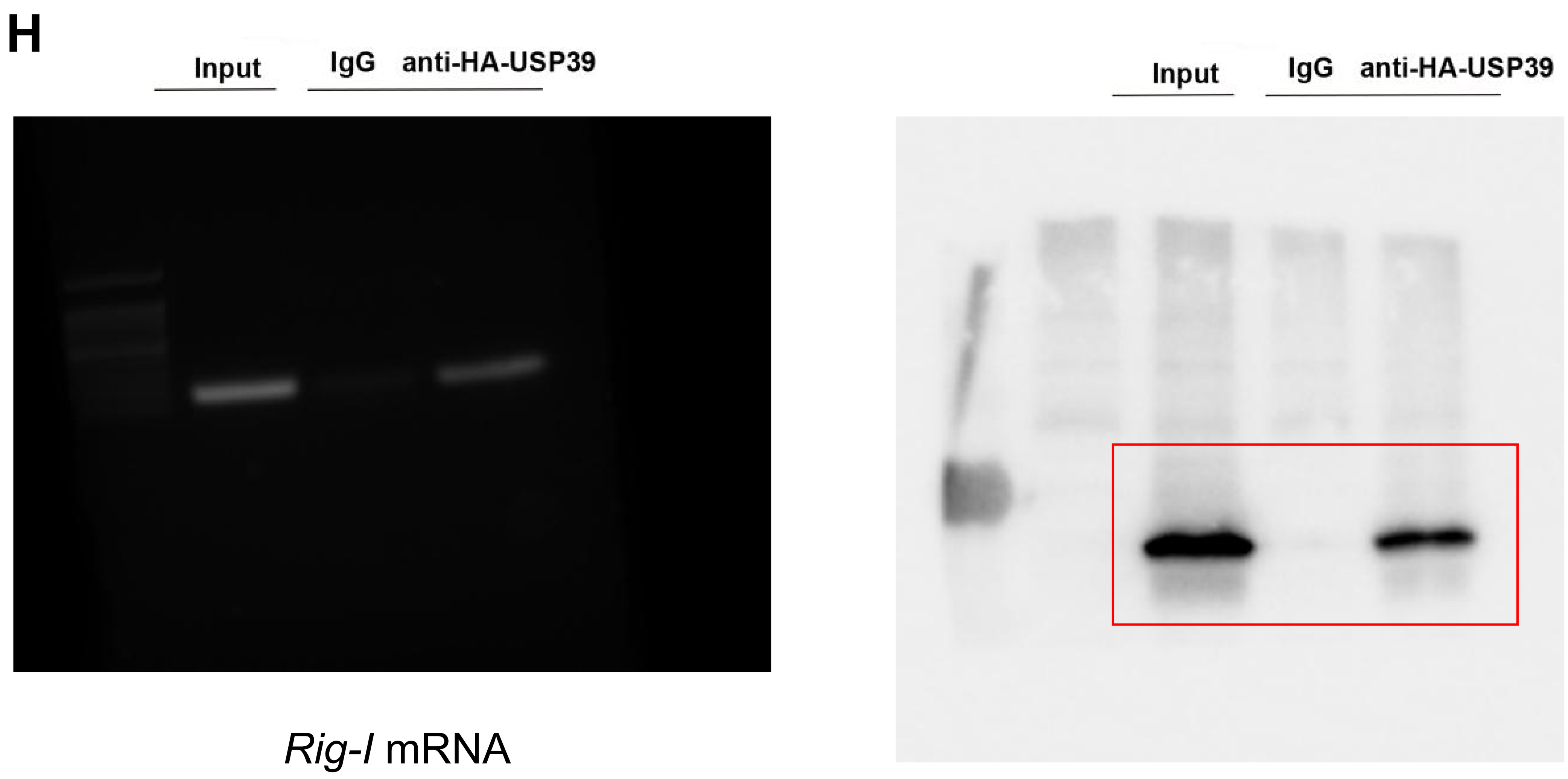

Rig-I mRNA

HA

Figure 6

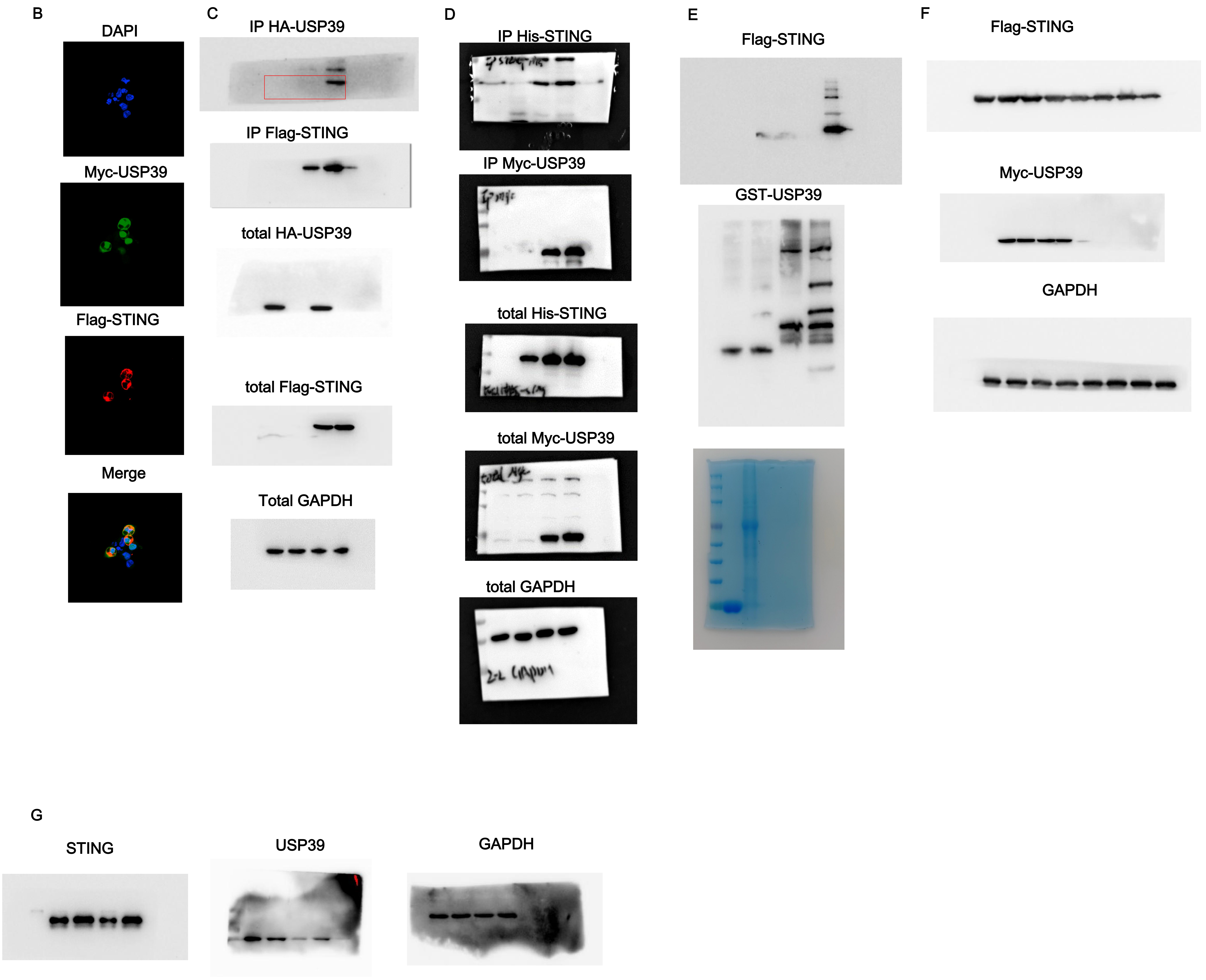
B
DAPI
Myc-USP39
Flag-STING
Merge
C
IP HA-USP39
IP Flag-STING
total HA-USP39
total Flag-STING
Total GAPDH
D
IP His-STING
IP Myc-USP39
total His-STING
total Myc-USP39
total GAPDH
E
Flag-STING
GST-USP39
F
Flag-STING
Myc-USP39
GAPDH
G
STING
USP39
GAPDH

Figure 6

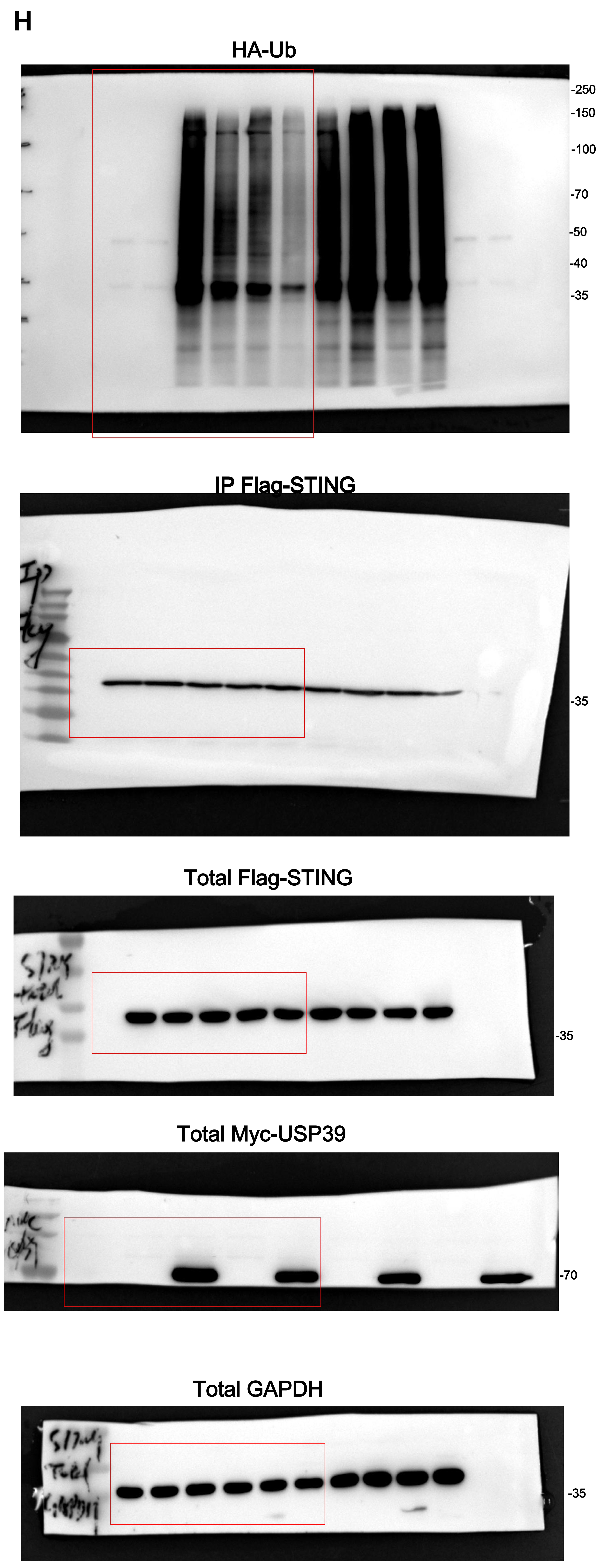
H
HA-Ub
-250
-150
-100
-70
-50
-40
-35
IP Flag-STING
-35
Total Flag-STING
-35
Total Myc-USP39
-70
Total GAPDH
-35

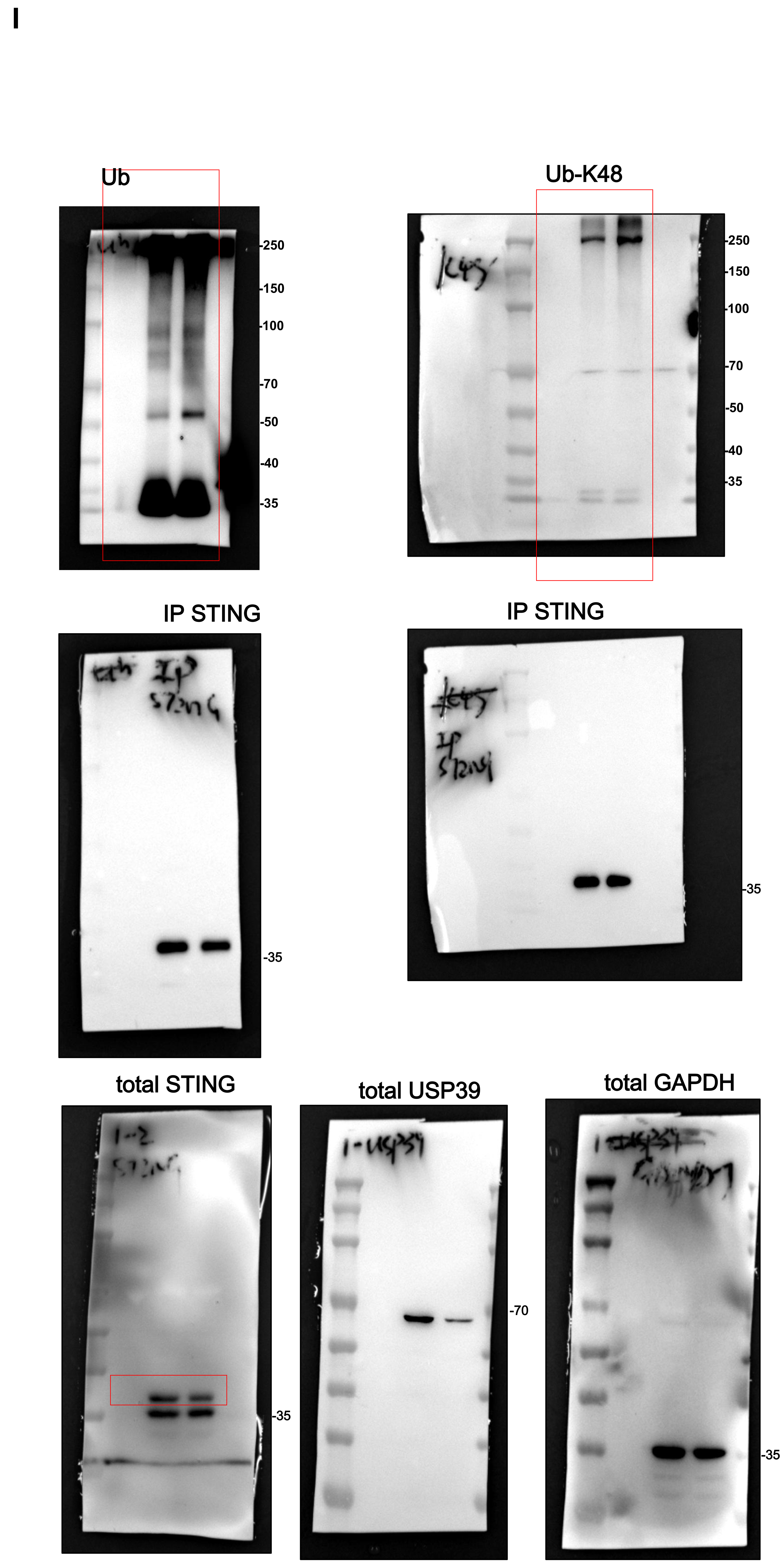
I
Ub
-250
-150
-100
-70
-50
-40
-35
Ub-K48
-250
-150
-100
-70
-50
-40
-35
IP STING
-35
IP STING
-35
total STING
-35
total USP39
-70
total GAPDH
-35

Figure 7

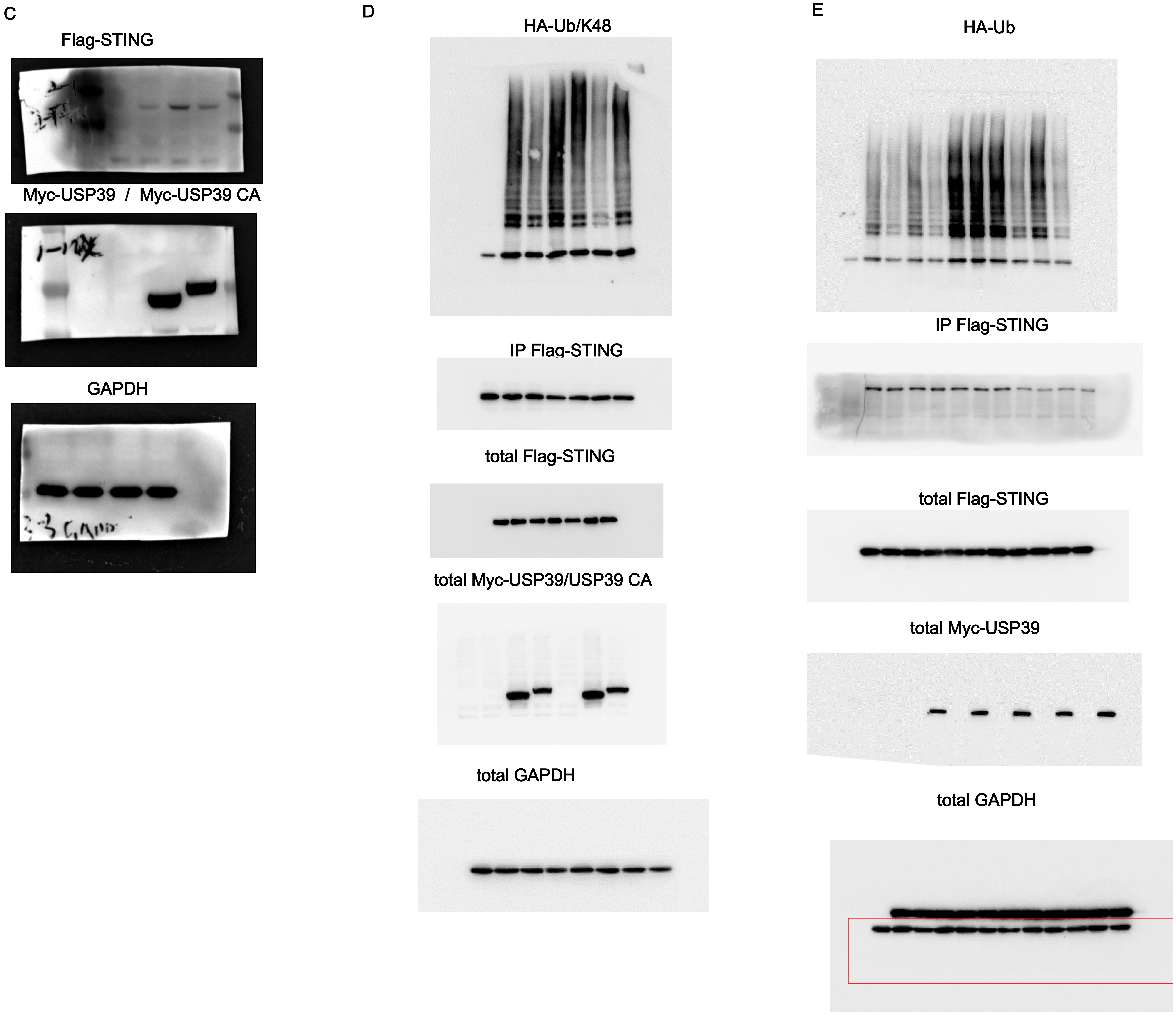
C
Flag-STING
Myc-USP39 / Myc-USP39 CA
GAPDH
D
HA-Ub/K48
IP Flag-STING
total Flag-STING
total Myc-USP39/USP39 CA
total GAPDH
E
HA-Ub
IP Flag-STING
total Flag-STING
total Myc-USP39
total GAPDH

Supplemental Figure 1

A

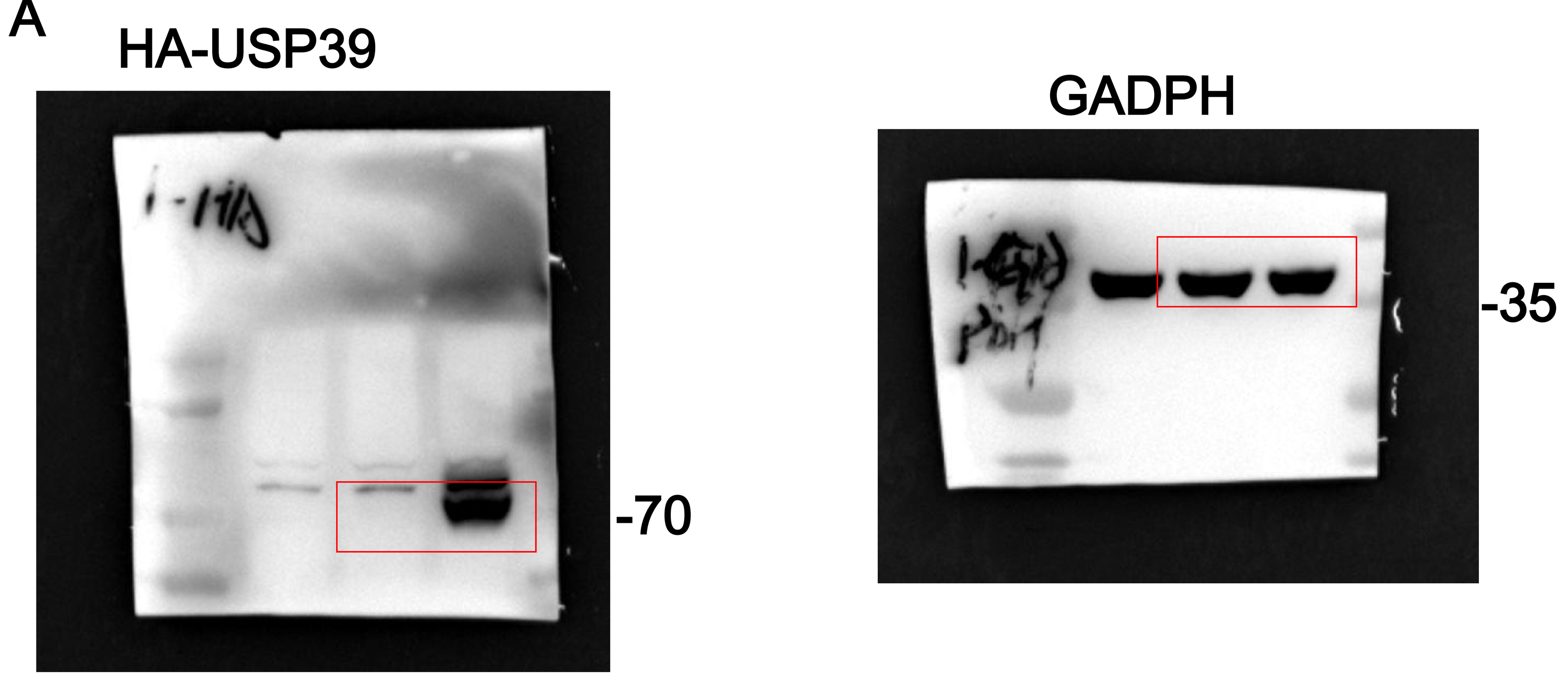

B

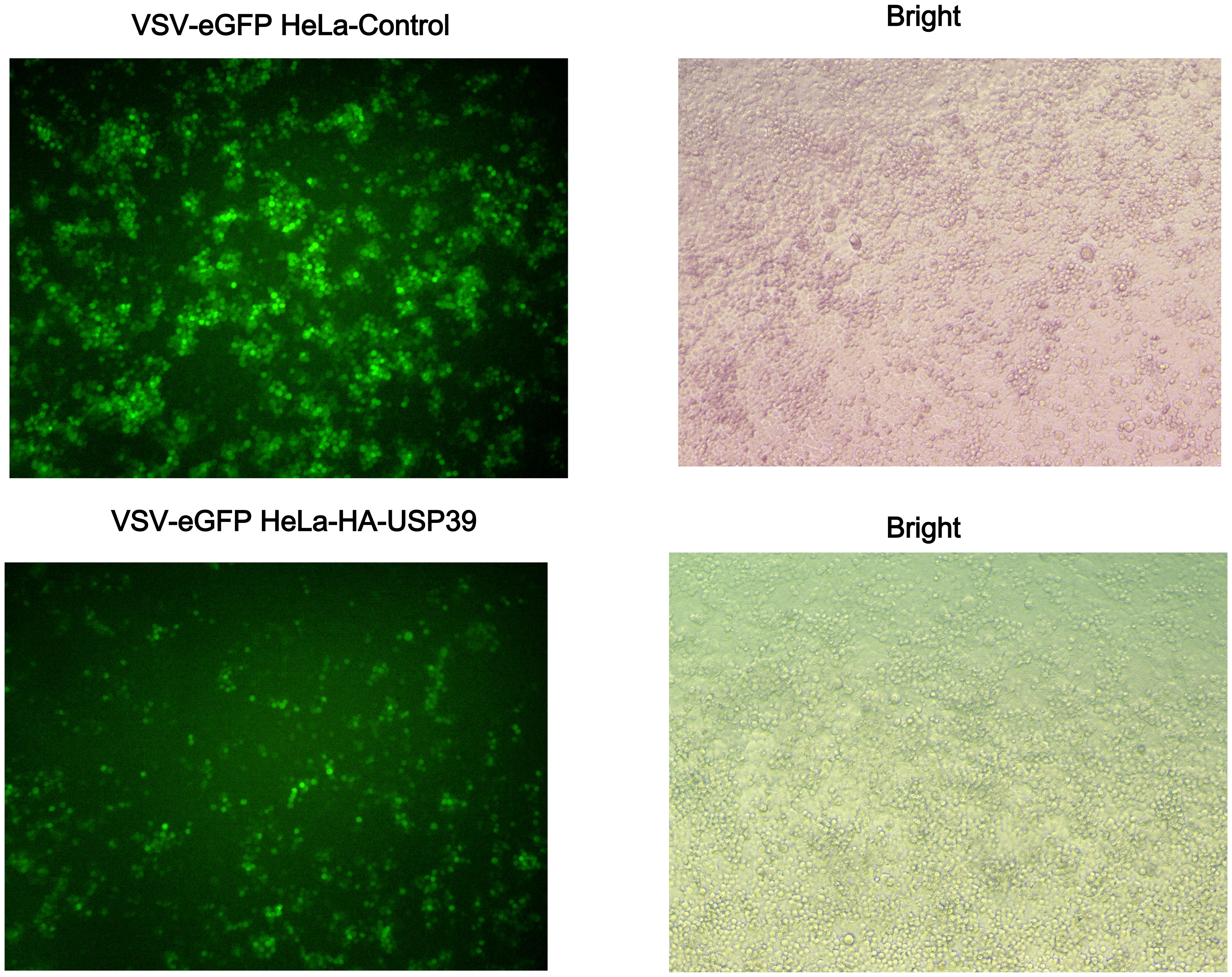

H

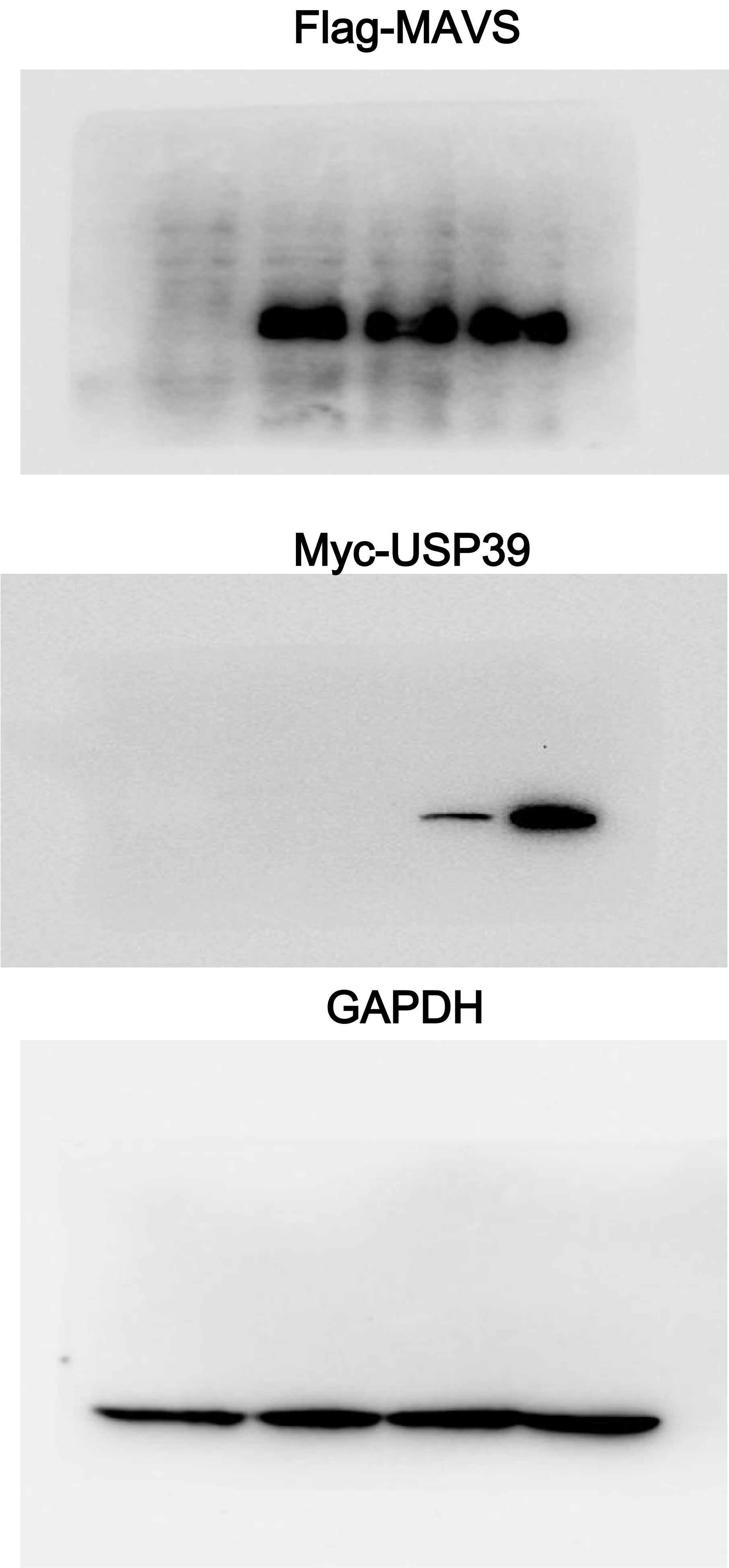

Supplemental Figure 2

G

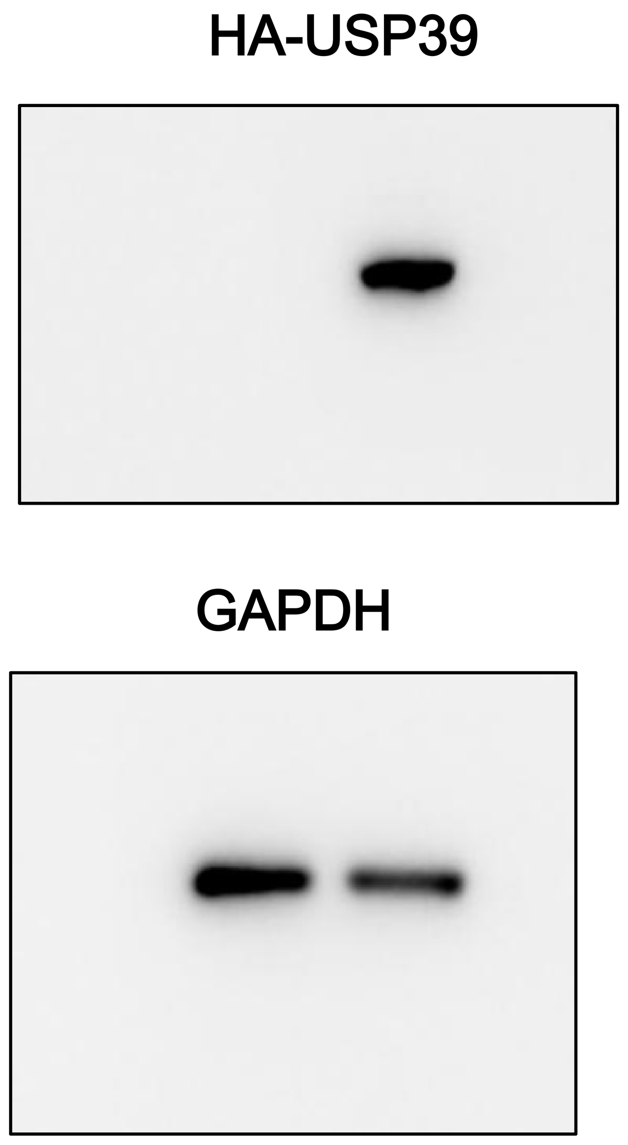

I

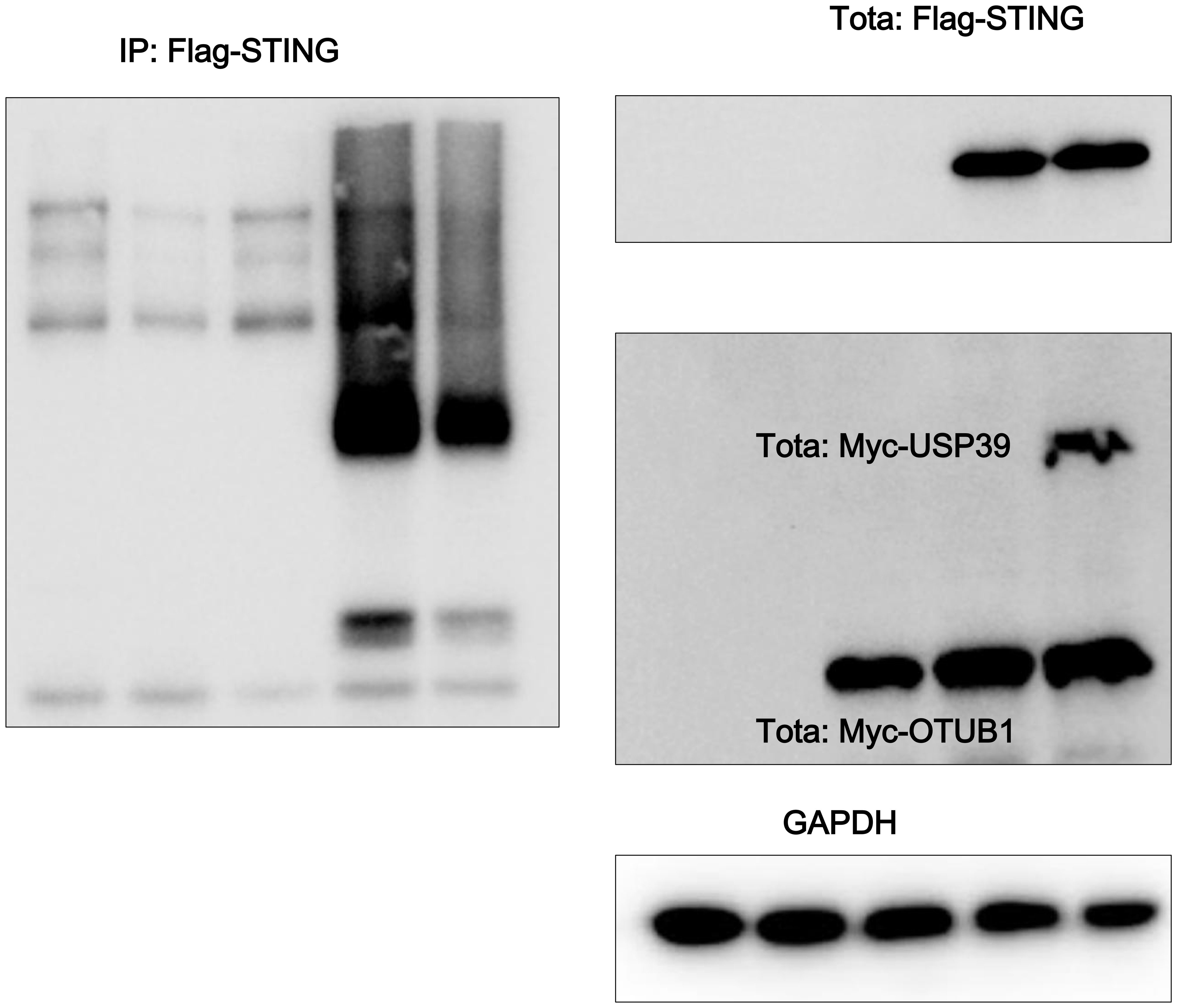

Supplement: S1 Raw Images — (PDF) [file pbio.3003796.s006.pdf]
