# Supplementary material for: USP39 promotes antiviral defense through post-transcriptional control of RIG-I and stabilization of STING
Source: PLoS Biol. 2026 May 11;24(5):e3003796. doi: 10.1371/journal.pbio.3003796 (PMC13178990; doi:10.1371/journal.pbio.3003796)
Supplement: Attachment — Submitted filename: 20251207 USP39-p-to-p.docx [file pbio.3003796.s007.docx]

> Reviewer #1:
>
*> The RIG-I/MAVS and cGAS/STING pathways play crucial roles in innate immune defence against viral pathogens but can also cause or contribute to detrimental inflammation. Many regulatory mechanisms have evolved in both pathways. This manuscript adds to the field's growing understanding of such mechanisms. Figs 1-3 nicely show in different in vitro and in vivo settings that USP39 positively controls innate immunity, particularly type I interferon induction, during RNA and DNA virus infection. The second part of the manuscript then dissects the underlying mechanisms. The authors claim that USP39 regulates RIG-I pre-mRNA splicing and STING ubiquitination. This is potentially of high interest because the same protein (USP39) controls different innate immune sensing pathways using very different molecular mechanism. This insight is not only of fundamental importance to our understanding of how innate immunity is controlled but potentially also relevant to targeting RIG-I and STING signalling for therapy. However, in my view, the data in Figs 4-7 are unconvincing and do not support the mechanistic claims well. I hope the authors will find my suggestions useful in further testing their model.*
**Response:** We sincerely appreciate your insightful comments and suggestions. Those comments are all valuable and very helpful for revising and improving our manuscript. According to your comments, we have performed additional experiments to support the mechanistic claims well and carefully revised the manuscript. In our revised manuscript, we added some new figures to strengthen our conclusion in Figs 4-7, including Figure S1H, Figure S1I, Figure 4C, Figure 4H, Figure 4I, Figure S2E, Figure 5H, Figure 6G. We believe these evidences can strongly support the conclusion that USP39 regulates antiviral immunity through dual mechanisms of promoting RIG-I pre-mRNA splicing and STING deubiquitination. Now we are submitting our revised manuscript, which we hope to address all the concerns.

*> Major points
> 1. Fig 3, HSV-1 infection: did the authors analyse* *viral replication, survival and/or pathology (as presented for VSV infections)?*

**Response:** Thanks for your valuable suggestion. Following your suggestions, we detected the IFN-β production, viral replication, survival and pathology after HSV-1 infection. The results also showed that Usp39 deficiency significantly inhibited IFN-β production, increased virus replication in the spleen, liver, and lung, exhibited lower survival rate and more severe pathological damage after HSV-1 infection. We have added the new results in Figure 3M-R in our revised manuscript (please see page 8, line 192-199).

*> 2. Fig 4:
> a. In Fig 4a and b, it appears MAVS is expressed at lower levels in USP39-deficient cells. Did the authors observe this effect reproducibly, and if so, how does this impact the interpretation of the results?*

**Response:** Thanks for your insightful comment. Indeed, the previous Figure 4A and B, it seems that MAVS expression is slight lower in USP39-deficient cells. To confirm these results, we repeated this experiment, and the new data showed that USP39 did not affect the protein level of MAVS. In addition, western blotting also revealed that overexpression of different doses of USP39 did not influence the protein level of MAVS in 293T cells. To further confirm this result, we detected the MAVS mRNA expression, and the data demonstrated that USP39 did not regulate the mRNA expression of MAVS. So, the data from new experiments indicate that USP39 does not regulate the expression of MAVS. We have replaced the previous data in new Figure 4A and B and added the new results in Figure S1H and Figure S1I. (please see page 8, line 211-218).

*> b. The experiments shown in* *Fig 4c and h would benefit from including cells that* *endogenously express RIG-I and STING. In addition, a negative control is missing: please include another Flag-tagged protein that is* *unaffected by USP39 overexpression.*

**Response:** Thanks for your suggestion. Following your advice, we added the endogenously express RIG-I and STING in new Fig 4C and Figure 4H, and the results also showed that USP39 increased the endogenously RIG-I and STING protein level. Moreover, we added the Flag-TBK1 as a negative control in new Fig 4I, which indicated that Flag-TBK1 was unaffected by USP39 overexpression. We have added the new result in our revised manuscript (please see page 8, line 219-220, page 9, and line 231-235).

*> 3. Fig 5 and S2 (RIG-I splicing):
> a. Fig S2a,b: For clarity, please indicate* *(using arrows) the positions of the primers* *used for RT-qPCR.*

**Response:** Thanks for your suggestion. We had added the primers positions information for RT-qPCR in new Figure S2A and B in our revised manuscript.

*> b. The mouse RIG-I gene has 18 exons. Why did the authors choose to study* *exons 2-3 only? Other exons and introns should be included to* *strengthen the conclusions.*

**Response:** Thanks for your valuable comment and suggestion. The mouse RIG-I gene has 18 exons, we randomly selected exons 2-3 for detection in our previous manuscript. Following your suggestion, we detected all the 18 exons and introns using RT-qPCR, and the data demonstrated that the ratio was significantly decreased in USP39-deficient cells, and strengthened our conclusions that USP39 was involved in RIG-I gene splicing (please see new Figure S2E, page 10, line 259-263).

*> c. Fig 5h / lines 245-250. How was RIG-I overexpressed in this setting? Did the authors transfect a RIG-I encoding plasmid? If yes, did this plasmid include the entire RIG-I gene or only the RIG-I* *cDNA? I could not find information in the methods regarding this point. Assuming a RIG-I cDNA plasmid was used here, the data are misinterpreted and would only show USP39 binding to* *mature RIG-I mRNA. However, the authors conclude that USP39 binds the pre-mRNA and allows mRNA maturation. Please clarify. Please also provide protein /* *western blot data to* *validate the IP and explain why* *both PCR and qPCR were used here.*

**Response:** We sincerely appreciate your insightful comment and thank you for pointing out our mistake in Figure 5H legend description and manuscript. As you said, the plasmid only contains the cDNA and mature mRNA sequence, and thus is not suitable for pre-RNA splicing assays. Actually, we only transfected HA-USP39 plasmid in the experiment and detected the endogenous pre-*Rig-i* mRNA. However, due to an oversight, an mistake was present in the figure legend description and manuscript. We have corrected it in the revised manuscript (please see page 10, line 265-266, and page 30, line 895-898). In addition, we added the western blot data of HA-USP39 to validate the IP in new Figure 5H. For using both PCR and qPCR to validate that USP39 bound to pre-*Rig-i* mRNA, we hope to confirm the result using different methods to enhance the reliability of the conclusion.

*> d. Line 260/261: these conclusions appear overstated. An alternative scenario is that USP39 has an impact of RIG-I pre-mRNA and/or mature mRNA stability.*

**Response:** Thanks for your valuable comment. According to your suggestion, we replaced the description of conclusion as “These results demonstrate that USP39 promotes RIG-I pre-mRNA splicing and maturation” in our revised manuscript (please see page 11, line 285-286). Moreover, we have added relevant discussion (Although our results demonstrate that USP39 could regulate the RIG-I pre-mRNA splicing and maturation, it was possible that USP39 also had other functions to regulate the expression of RIG-I, such as promoting mature RIG-I mRNA stability) in our revised manuscript(please see page 14, line 381-384).

*> e. Have the authors attempted northern blotting to demonstrate defects in RIG-I pre-mRNA processing using an alternative method (not involving amplification)?*

**Response:** Thanks for your comment and suggestion. There are many experimental techniques for detecting protein-RNA binding, as you suggested, northern blotting is indeed a classic method. However, with the development of diversified detection methods in recent years, RT-qPCR and RIP (RNA Immunoprecipitation) followed by PCR amplification is commonly used to demonstrate protein-RNA binding at the present (PMID: 37450113). As a result, experimental platforms equipped to perform northern blotting have become relatively scarce and often not fully optimized. Our laboratory, in particular, lacks the necessary conditions to perform northern blotting, and therefore we were unable to perform related detection using this technique.

To further confirm that USP39 binds to RIG-I pre‑mRNA, we repeated RT-qPCR and RIP experiments, and also detected the regulatory effect of USP39 on all 18 exons of RIG-I. The results confirmed that USP39 could bind to RIG-I pre-mRNA and promote its splicing and maturation (please see new Figure S2E). We hope these reliable results can address your concerns.

*> f. Line 348. The authors must cite and* *discuss this article on a RIG-I splice variant: pmid: 18948594.*

**Response:** Thanks for your comment. According to your suggestion, we had cited and discussed the article on RIG-I splice variant , and added the discussion “The intact tandem CARD domains of RIG-I are crucial for its function. Its splice variant (SV), which harbors a short deletion of amino acids 36–80 within the first CARD domain, lacks the ability to bind TRIM25, the capacity for CARD domain ubiquitination, and downstream signal transduction capability” (please see page 14, line 367-371, ref 57).

*> 4. There are a number of technical concerns regarding Figs 6 and 7 (STING deubiquitination):
> a. Fig 6c should include* *an extra negative control: e**xpression of Myc-USP39 alone, followed by anti-FLAG immunoprecipitation.*

**Response:** Following your suggestion, we had added an extra negative control in new Figure 6C of our revised manuscript, only expression of Myc-USP39, and followed by anti-FLAG immunoprecipitation.

*> b. Fig 6e should include* *an extra negative control:* *pulldown using GST and Flag-STING to exclude* *non-specific binding of STING to the beads.*

**Response:** Thank you for your valuable suggestion. In new Figure 6E of our revised manuscript, we have added an additional negative control, pulldown using only GST and Flag-STING, to exclude the possibility of non-specific binding of STING to the beads.

*> c. Fig 6f: comparing lanes 3 and 4, there is a strong reduction in the Flag-STING signal; however, this is not reflected in the* *quantification below the blots. Moreover, for the quantification, the data from the three independent repeats need to be shown together.*

**Response:** Thank you for your comment. To address your concern and confirm the result, we repeated the related experiment, and the result showed that USP39 overexpression could reliable stabilize and increase the half-life of STING. Following your advice, we re-analyzed the quantification of western blot data from three independent experiments and performed the statistical analysis, the results also showed that USP39 overexpression significantly increased the half-life of STING (please see new Figure 6F).

> *d. Fig 6g should include* *an extra control:* *MG132 treatment of USP39-sufficient cells to exclude non-specific binding of MG132.*

**Response:** Thanks for your valuable comment. According to your suggestion, we added a MG132 treatment of USP39-sufficient cells group as an extra control in new Figure 6G. The data revealed that MG132 treatment could rescue USP39 deficiency-caused STING degradation. In addition, to confirm whether USP39 regulates the half-life of endogenous STING, we use CHX to treat macrophages. The result showed that USP39 could obviously increase the half-life of endogenous STING (please see new Figure 6G).

*> e.* *Fig 7d: The authors conclude that the* *catalytically dead USP39 is not able to de-ubiquitinate STING. However, comparing the HA signal in Flag-IPs (lanes 4 vs 5 and lanes 7 vs 8),* *differences appear marginal. Have the authors quantified results from multiple independent experiments?*

**Response:** Thanks for your valuable comment and suggestion. Our study demonstrated that USP39, not the catalytically dead USP39, could mediate deubiquitination of STING, but it seemed that the differences appear unapparent in our previous Figure 7D. To address your concern and confirm the result, we repeated the related experiment, and the result showed that USP39, but not the catalytically dead USP39, could obviously mediate deubiquitination of STING in lanes 4 vs 5 and lanes 7 vs 8. As ubiquitination modification often generates a smeared pattern instead of a single sharp band on western blots, so precise quantification becomes challenging. Therefore, we used the new Figure 7D exhibiting a significant difference instead of the previous data, to ensure a clear and unambiguous interpretation.

*> 5.* *PMID 33127822* *suggests that USP39 regulates STAT1. The authors should* *exclude that* *the effects observed here are an indirect consequence of USP39 facilitating IFN-IFNAR-JAK/STAT signalling, perhaps* *at baseline. In particular, the RIG-I gene is IFN-inducible and controlled by STAT1. Key experiments should be repeated in STAT1 KO cells.*

**Response:** Thanks for your valuable suggestion. The previous study (PMID 33127822) suggests that USP39 can deubiquitinate and stabilize STAT1. To exclude the effects of USP39 on IFN-IFNAR-JAK/STAT signalling. We get the *Stat1*-knockout cells, and the results showed that USP39 promoted VSV-induced IFNb expression and RIG-I pre-mRNA splicing (at baseline) in a STAT1 independent manner(please see page 11, line 279-285, new Figure 5M, Figure 5N, Figure S2F and Figure S2G).

*> 6. Many western blots lack* *size markers. Please add these to all* *western blots shown in main and supplementary figures.*

**Response:** Thank you for your comment. Following your suggestion, we had added size markers in all western blots shown in main and supplementary figures.

*> 7. The manuscript requires substantial English language editing.*

**Response:** Thank you for your comment and suggestion. We engaged a native English-speaking expert (Scientific Editor, Shenzhen University School of Medicine) to perform the language polishing on the revised version, ensuring that the manuscript meets the linguistic standards of international academic journals. These revisions will significantly enhance the readability and precision of scientific expression in the manuscript.

*> Minor points
> 1. Line 27: RIG-I is not an adaptor. It is an RNA sensor. The adaptor protein in the pathway is MAVS.*

**Response:** Thank you for pointing out our mistake. Following your suggestion, we have revised the description in the abstract (please see page 2, line 26).

*> 2.* *References 10, 16, 19, 27 and 36 are very old. Please* *replace with more recent reviews.*

**Response:** Thanks for your valuable suggestion. We have replace the old references with more recent reviews in our revised manuscript (please see new ref 10, 16 , 17 , 28 and 38).

*> 3. Line 81: If* *citing ref 18 (describing the discovery of MAVS), please also cite the three other papers thar simultaneously discovered MAVS:
> a. Seth, R.B., Sun, L., Ea, C.K., and Chen, Z.J. (2005). Identification and characterization of MAVS, a mitochondrial antiviral signaling protein that activates NF-kappaB and IRF 3. Cell 122, 669-682. 10.1016/j.cell.2005.08.012.*
*> b. Meylan, E., Curran, J., Hofmann, K., Moradpour, D., Binder, M., Bartenschlager, R., and Tschopp, J. (2005). Cardif is an adaptor protein in the RIG-I antiviral pathway and is targeted by hepatitis C virus. Nature 437, 1167-1172. 10.1038/nature04193.
> c. Xu, L.G., Wang, Y.Y., Han, K.J., Li, L.Y., Zhai, Z., and Shu, H.B. (2005). VISA is an adapter protein required for virus-triggered IFN-beta signaling. Molecular cell 19, 727-740. 10.1016/j.molcel.2005.08.014.*

**Response:** Thank you for your suggestion. In addition to citing reference 18, we have also cited the three related papers (PMID: 16125763, 16177806, and 16153868) that simultaneously reported the discovery of MAVS in the revised manuscript (please see ref 20, 21 and 22).

*> 4. Line 88.* *Alongside ref 28, this paper also needs to be* *cited here: Wu, J., Sun, L., Chen, X., Du, F., Shi, H., Chen, C., and Chen, Z.J. (2013). Cyclic GMP-AMP is an endogenous second messenger in innate immune signaling by cytosolic DNA. Science 339, 826-830. 10.1126/science.1229963.*

**Response:** Following your suggestion, we have cited the paper (PMID: 23258412, Wu, J., Sun, L., Chen, X., Du, F., Shi, H., Chen, C., and Chen, Z.J. (2013). Cyclic GMP-AMP is an endogenous second messenger in innate immune signaling by cytosolic DNA. Science 339, 826-830. 10.1126/science.1229963) in the revised manuscript (please see new ref 30).

*> 5. Line 137: the term "**immunofluorescence" appears to be incorret here, unless the GFP signal was detected using an anti-GFP antibody.*

**Response:** Thank you for your comment and pointing out our mistake. We have replaced “by immunofluorescence assay” to “by fluorescence microscopy” in the revised manuscript (please see line 133).

*> 6. The* *p-TBK1 blot in Fig 4d is very dark. Can the authors* *provide a shorter exposure?*

**Response:** Thanks for your valuable comment. According to your suggestion, we have provided a shorter exposure p-TBK1 blot in new figure 4D.

*> 7. Lines 230-232: I suggest replacing* *"was also downregulated" with "showed reduced induction" to better represent the data:* *USP39-deficient cells still show an increase in RIG-I mRNA levels after infection or pI:C transfection.*

**Response:** Thanks for your valuable suggestion. In accordance with the advice, we have modified the relevant descriptions in the manuscript to improve clarity and accuracy (please see line 245-247).

*> 8.* *Please* *replace "Extron" with "Exon" in the text and figures.*

**Response:** Thank you for your suggestion. We have replaced "Extron" with "Exon" in the text and figures.

*> 9. Line 487. Please include a* *reference to the* *publication where these steps were described.*

**Response:** Thanks for your valuable suggestion. We have added the reference to describe these steps form the publication in the revised manuscript (please see ref 60).

*> 10. Line 806: typo (STIGN)*

**Response:** Thank you for pointing out our mistake. We have revised the spelling mistake (Flag-STING) in line 917.

*> Reviewer #2:*

*>
> This manuscript by Quan et al. reports that USP39 promotes anti-viral responses via RIG-I and cGAS-STING to suppress viral replication in cultured cells (murine macrophages and HeLa cells) and in vivo. Using Usp39fl/fl Lyz2-Cre macrophages, the authors demonstrate that USP39 plays an important role in limiting both RNA and DNA viral infection. They further provide evidence that this is through USP39's role in promoting type-I interferon anti-viral responses. Consistently, mouse infection models (VSV and HSV-1) reveal a similar role for USP39 in macrophages in promoting type-I IFN responses to limit viral replication in vivo through analysis of spleen, liver and lung of the mice. Mechanistically, the study provides evidence that USP39 positively regulates RIG-I levels by facilitating splicing of Rig-i pre- mRNA into the mature mRNA. The study claims that USP39 positively regulates the cGAS-STING pathway by stabilising STING through deubiquitylation of Lysine288 and thus preventing proteasomal degradation.*
**Response:** Thanks for your valuable comments.

*> Overall, the study provides novel insights into the role of USP39 in protective anti-viral responses to both RNA and DNA viruses. The data in the manuscript nicely supports this conclusion. The data underpinning the mechanistic aspects of the study is somewhat less convincing and it is not clear why the study* *focusses specifically on RIG-I and STING as the targets of USP39 - might USP39 not regulate other components of the pathways? The proposed* *role for USP39 in regulating ubiquitylation of STING needs to be further investigated as the used methodologies to study this are suboptimal and could lead to wrongful conclusions. Specific comments are listed below.*
**Response:** We sincerely appreciate your insightful comments and suggestions. Those comments are all valuable and very helpful for revising and improving our manuscript. In this study, we analyzed the key proteins of RIG-I-MAVS signaling pathway activated by RNA virus infection and cGAS-STING signaling pathway activated by DNA virus infection via western blot, and found that USP39 could specifically regulate the expression of RIG-I and STING (please see new Figure 4A and B). So, in subsequent studies, we mainly focused specifically on RIG-I and STING as the targets of USP39. According to your comment, we have performed additional experiments to demonstrate the role for USP39 in regulating deubiquitylation of STING and carefully revised the manuscript. In our revised manuscript, we added new figures to strengthen our conclusion in including Figure S1H, Figure S1I, Figure 4C, Figure 4H, Figure 4I, Figure S2E, Figure 5H, Figure 6G. We believe these evidences can strongly support the conclusion that USP39 can regulate STING deubiquitination. Now we are submitting our revised manuscript, which we hope to address all the concerns.

*> Main Points:
> 1. Are the authors sure that USP39-regulation of RIG-I levels is the only mechanism by which USP39 regulates the pathway? In Fig 4B MAVS levels also appear reduced in USP39 KO macrophages. Does USP39 also affect splicing of the pre-mRNA encoding MAVS or does it regulate MAVS stability as proposed for STING?*

**Response:** Thanks for your insightful comment. Indeed, the previous Figure 4B, it seems that MAVS expression is slight lower in USP39-deficient cells. To confirm these results, we repeated this experiment, and the new data showed that USP39 did not affect the protein level of MAVS. In addition, western blotting also revealed that overexpression of different doses of USP39 did not influence the protein level of MAVS in 293T cells. Furthermore, to confirm this result, we also detected the MAVS mRNA expression, and the data demonstrated that USP39 did not regulate the mRNA expression of MAVS. So, our new experiments indicate that USP39 does not regulate the expression of MAVS. We have replaced the previous data in new Figure 4A and B and added the new results in Figure S1H and Figure S1I. (please see page 8, line 211-218).

*> 2. Fig 5 I-J. Th**e authors should* *determine how the re-expression of USP39 impact on splicing of Rig-i mRNA in the macrophages to substantiate their proposed mechanism. Comparison with the USP39-CA in these cells will strengthen their conclusions. The effect of USP39 and USP39-CA on* *Rig-i mRNA in Figure 5K supports their model but also in these cell model, the effect on Rig-i splicing should be evaluated.*

**Response:** Thanks for your valuable comment. According to your suggestion, we have added the experiments to determine whether re-expression of USP39 impacted on splicing of *Rig-i* pre-mRNA in USP39-deficient macrophages. The results showed that re-expression of USP39 in USP39-deficient macrophages obviously promoted *Rig-i* pre-mRNA splicing and maturation (please see new Figure 5K). In addition, except detecting the expression of *Rig-i* mRNA in USP39 and USP39-CA overexpression cells, we also added the experiments to confirm whether overexpression of USP39 and USP39-CA impacted on splicing of *Rig-i* pre-mRNA. The results revealed that USP39 and USP39-CA overexpression could both significantly promoted *Rig-i* pre-mRNA splicing and maturation, suggesting that USP39 regulated *Rig-i* pre-mRNA splicing and maturation independent on its deubiquitinase activity (please see new Figure 5L, please see page 10, line 273-275, and page 11, line 279-280).

*> 3. The authors claim that USP39* *counteracts K48-linked ubiquitination of STING but the experiments shown do not prove this since they IP STING and blot for ubiquitin (total or HA-Ub or K48-Ub). Since IP's are done under non-denaturing conditions, it is not possible to know if the ubiquitin smears observed are* *conjugated to STING or to other proteins associated with STING. Instead, the experiments should be repeated so* *ubiquitin is enriched by IP or TUBE pulldown, followed by SDS-PAGE and blotting for STING. If STING migrates as a smear in SDS-PAGE it would strongly indicate that STING is covalently modified with ubiquitin. To further prove that the smear is due to STING ubiquitylation the ubiquitin-enriched sample should treated with recombinant deubiquitinases (e.g. USP21 or OTUB1 (K48-selective)) which will result in a collapse of the smeared STING signal to a single band. The analysis of STING ubiquitination in Figure 7 should also be repeated as described above.*

**Response:** Thanks for your insightful comment and suggestion. As your said, non-denaturing IP experiment is used to demonstrate protein-protein interaction. So there may be some interacting proteins to conjugate the IP target protein. However, in our ubiquitination assay, we performed the IP according to the denatured IP assay protocol. Briefly, for ubiquitination assay, cells were lysed in IP buffer (containing 1% of SDS and a 1 × protease inhibitor mixture) and boiled for 5min at 95 ℃. Supernatant was collected and diluted 10-fold in IP buffer, following IP with indicated antibody for 2 hours, and then incubated with protein A/G Plus-Agarose or Anti-Flag M2 magnetic beads at 4°C for 12 h. The samples were then washed in immunoprecipitation buffer five times, and the beads were diluted in SDS buffer and boiled for 10 min. Equal amounts of protein were analyzed by western blotting, as described previously (PMID: 28878677). We have added these process in the Ubiquitination assay of Materials and Methods in our revised manuscript (please see page 18, line 497-504). Therefore, in our ubiquitination assay, we performed the denatured IP assay protocol and the ubiquitin smears observed was the STING ubiquitination.

Moreover, according to your suggestion, we have added the experiments that HA-Ub-K48 is enriched by IP, and followed by blotting and SDS-PAGE for detecting STING. The result demonstrated that not only the ubiquitination of STING (the upper band in IP group), but also the STING with no ubiquitination modification (the below band in IP group), were both decrease. These data strongly demonstrate that USP39 counteracts K48-linked ubiquitination of STING (please see new Figure S2H).

Furthermore, previous studies have shown USP21 or OTUB1 (K48-selective) was important deubiquitinating enzymes to remove the ubiquitination modification of the substrate protein. We have cited the related references in our revised manuscript (please see new ref 64 and 65). In addition, the analysis of STING ubiquitination in Figure 7 also used the denatured IP assay.

*> 4. The authors conclude that USP39 deubiquitinates K288 on STING to stabilise the protein but the reasoning for this interpretation is not clear. As pointed out by the authors, the ubiquitination signal in Fig 7E is stronger in the samples with K288R STING than in WT STING or the other K>R mutants. However, if K288 is a major ubiquitylation site on STING that is regulated by USP39, the ubiquitination of this STING mutant should be reduced and not increased. Alternatively,* *if STING is modified on multiple sites, the apparent "length" of the smear should be shorter as the total number of Ub molecules conjugated to each STING K288R molecule would be less than for WT STING. Also, to evaluate the effect of USP39 on the* *ubiquitination of different STING* *mutants, the ubiquitination of these should be shown with and without co-expression of USP39. This would enable an assessment of the effect of USP39 expression rather than an assessment of the K>R mutation itself.*

**Response:** Thanks for your insightful comment and suggestion. Our study revealed that USP39 deubiquitinates K288 on STING to stabilize the protein. According to these results, we hypothesis that STING ubiquitination was modified on multiple sites and by many E3 ubiquitin ligases and deubiquitinating enzymes, and in a state of dynamic equilibrium. So, one of STING ubiquitination site mutant may affect other STING ubiquitination sites modification, and the interaction or/and function of other E3 ubiquitin ligases and deubiquitinating enzymes. Therefore, we observed the results that the ubiquitination signal in Fig 7E was stronger in the samples with K288R STING than in WT STING or the other K>R mutants.

In accordance with your suggestion, to evaluate the effect of USP39 on the different STING mutants ubiquitination modification. We repeated the ubiquitination assay using various ubiquitination site mutant STING, with and without co-expression of USP39. The result confirmed that USP39 deubiquitinates STING depending on K288 lysine site of STING (please see new Figure 7E).

*> Specific Points:
> 1. Authors should define which murine macrophages are used in their study. Are these BMDMs?*

**Response:** Thanks for your valuable suggestion. In most experiments, we used macrophages were isolated from the abdominal cavity of mice through intraperitoneal injection thioglycollate broth. In some overexpression experiments (using lentivirus for pre-infection), we used BMDMs. We have added these experimental information in the Materials and Methods in our revised manuscript (please see page 19, line 533-537).

*> 2.* *In Fig 5 and accompanying text, the authors use the word "Extrons" to describe what I presume are spliced exon-exon boundaries in the* *mature mRNA or do they refer to refer to something else. Please clarify.*

**Response:** Thanks for your valuable suggestion. Following your advice, in Fig 5 and accompanying text, we have changed the describe in the mature mRNA or pre mRNA (please see page 9-10, line 248-250, and new Figure 5F, 5G, 5H, Figure S2C and S2D).

*> 3.* *Figure 1H/1K. The effect of* *USP39 KO on IFNbeta production* *in response to VSV and poly(I:C) is* *relatively mild (10%-15% reduction), which could indicate that regulation of IFNbeta is not the main function of USP39.*

**Response:** Thanks for your insightful comment. In our original manuscript, the effect of USP39 KO on IFNbeta production seemed relatively mild in Figure 1H/1K. To confirm this result, we repeated these experiment, and the results showed that USP39 KO significantly inhibited IFNbeta production in response to VSV and poly(I:C). We have replaced these results with more reliable data in the revised manuscript (please see new Figure 1H and 1K).

*> 4. Line 249. It is unclear what "compounds" refers to.*

**Response:** Thanks for your comment. To make the description clearer, we have changed the describe to “*RIG-I* pre-mRNA was significantly enriched in USP39 pull-down assay” in the revised manuscript (please see page 10, line 268).

*> 5. Fig 6F: The quantification of the protein levels of Flag-STING is unconvincing and appears to contradict the western blots shown: Comparison of the Flag signal in lane 4 vs 1 shows a decrease in signal that is clearly more pronounced than in lane 8 vs 5. However, the graph shoes that USP39 stabilises STING. The quantification should be repeated multiple times and error bars should be included with statistical analysis. Also, the blots are overexposed, which is problematic when trying to quantify the signal.*

**Response:** Thank you for your comment. To address your concern and confirm the result, we repeated the related experiment, and the result showed that USP39 overexpression could reliable stabilize and increase the half-life of STING. Following your advice, we re-analyzed the quantification of western blot data from three independent experiments and performed the statistical analysis, the results also showed that USP39 overexpression significantly increased the half-life of STING (please see new Figure 6F).

*> 6. Fig 6G: MG132 treatment of Usp39^fl/fl^ cells should be included.* *CHX time course treatments should also be included to determine if, indeed, USP39 increases the half-life of endogenous STING.*

**Response:** Thanks for your valuable comment. According to your suggestion, we added a MG132 treatment of Usp39^fl/fl^ cells group as an extra control in new Figure 6G. The data revealed that MG132 treatment could rescue USP39 deficiency-caused STING degradation. In addition, to confirm whether USP39 regulates the half-life of endogenous STING, we use CHX to treat macrophages. The result showed that USP39 could obviously increase the half-life of endogenous STING (please see new Figure 6G).
