# Supplementary material for: USP39 promotes antiviral defense through post-transcriptional control of RIG-I and stabilization of STING
Source: PLoS Biol. 2026 May 11;24(5):e3003796. doi: 10.1371/journal.pbio.3003796 (PMC13178990; doi:10.1371/journal.pbio.3003796)
Supplement: Attachment — Submitted filename: 20260410 USP39-p-to-p.docx [file pbio.3003796.s008.docx]

Reviewer #1: 
*> The authors added a lot of new data to their manuscript and have addressed most of my comments adequately. However, I remain confused by some of the data, particularly the new data in Fig 4I. Here, the authors over-express using plasmid transfection RIG-I, STING and TBK1. The western blot shows that co-expression of HA-USP39 has no impact on TBK1 protein levels. This is a good control. In contrast, both RIG-I and STING protein levels are increased in HA-USP39 expressing cells. This supports the authors' model for STING (post-translation regulation) but not for RIG-I (regulation of splicing). Presumably, the RIG-I plasmid used here does not contain introns; therefore - if the proposed mechanism is correct - no effect at protein level would be expected in this setting. As such, I am regrettably left unconvinced regarding the mechanism(s) by which USP39 regulates RIG-I.*

**Response:** Thanks for your valuable comments, we fully agree with your point of the concern. In our study, we tried to demonstrate that USP39 positively regulates antiviral immunity by promoting RIG-I pre-mRNA splicing and STING deubiquitination. Indeed, it seems that both RIG-I and STING protein levels are increased in HA-USP39 expressing cells in Fig 4I, but the RIG-I plasmid does not contain introns. We are also puzzled by this result. To confirm this results, We repeated the experiment using Flag-RIG-I and HA-USP39 plasmids. The result reveal that overexpression USP39 indeed slightly increased RIG-I protein (as shown below).

We think there might be two possible reasons: (1) Since USP39 is a ubiquitin-specific peptidases, it may regulate the RIG-I deubiquitination and stabilization at the post-translational level, then promoting the increase of RIG-I. (2) As a regulatory factor that promotes RIG-I pre-mRNA splicing, USP39 does not separate from the mature mRNA after pre-mRNA splicing is completed. Instead, it continues to bind to the mature RIG-I mRNA and plays a role in stabilizing it (that is, USP39 not only bind to the pre-mRNA of RIG-I, but also to the mature mRNA of RIG-I), thereby promoting the increase of RIG-I.

So, in order to further verify the hypothesis, we first performed ubiquitination assays to detect the effect of Myc-USP39 on regulating Flag-RIG-I protein ubiquitination (MG132 treated, equal RIG-I protein amount). The results showed that USP39 could not regulate the ubiquitination level of RIG-I (New Figure 4J), indicating that USP39 regulates RIG-I not at the post-translational protein level. Furthermore, we co-overexpressed HA-USP39 and Myc-RIG-I plasmids in cells, and then performed RNA-binding protein immunoprecipitation (RIP) assays. The results demonstrated that the USP39 could also bind the mRNA of RIG-I (New Figure S2H), suggesting that USP39 might bind the mature RIG-I mRNA and plays a role in stabilizing it, then promoting the increase of RIG-I.

Collectively, combining the data in our manuscript (Figure 5A-H) with these new data (New Figure 4J and S2H) indicate that USP39 regulate RIG-I expression not only bind to the RIG-I pre-mRNA to promote RIG-I mRNA maturation, but also bind to the mature RIG-I mRNA to stabilize it. We have added the new results and discussion in our revised manuscript (please see Page 9, Line 235-240; Page 11, Line 284-289; Page 15, Line 398-409).
> 
> Reviewer #2:  *> The authors have addressed most of my concerns and have included in their revised manuscript added important additional experimental data to support their conclusions. However, they have not adequately addressed the concerns raised regarding the direct regulation of K48-liked ubiquitination of STING by USP39. The authors claim that their use of a denaturation IP protocol to evaluate STING ubiquitination proves that the ubiquitin signals are conjugated to STING, referring to one of their previous publications involving NEMO ubiquitination. While the denaturation protocol has been used by some groups to show ubiquitination of NEMO, this remains a contested topic because this is not reproduced when performing pulldown experiments of ubiquitin and blotting for NEMO. Likewise, purification of receptor signalling complexes did not reveal any extensive polyubiquitnation of NEMO. A likely explanation for the discrepancy is that the fact that ubiquitin is not denatured by 1% SDS
> and, secondly, that ubiqutin-binding domains renature to some extend when the SDS concentration is lowered so that Ub chains can be co-purified. 
> This may or may not be the case for STING but can the authors be sure that the Ub chains co-purified with STING indeed are conjugated to STING? As suggested previously, the authors could address this by treating their ubiquitin- or STING-enriched samples with recombinant deubiquitinases (e.g. USP21 or OTUB1 (K48-selective)) which will result in a collapse of the smeared STING signal to a single band (USP21) or lower the apparent MW of the "smears" (OTUB1) if the ubiquitin chains indeed are conjugated to STING. See ref:* *[https://www.nature.com/articles/nprot.2015.018](https://www.nature.com/articles/nprot.2015.018#/list/1/_blank) and related articles citing the paper. Along this line, it is curious that the ubiquitin smears in Fig. 7E all have the same migration pattern despite the mutation of specific ubiquitination sites on STING. Would this not suggest that the (majority of the) ubiquitin signals detected comes from ubiquitin chains not directly conjugated to STING? Irrespective, I strongly suggest to either perform the suggested*
*> experiments or alternatively, to include a brief discussion of the limitation of this part of the study.*

**Response:** Thanks for your valuable comments, we greatly appreciate your insightful comments and fully agree with your point of the concern. According to your suggestion, we got the Myc-OTUB1 expression plasmid. To verify whether the ubiquitin signals detected are covalently conjugated to STING rather than non-covalently associated ubiquitin chains, we performed co-expression assays in 293T cells by transfecting HA-Ub, Flag-STING, and Myc-OTUB1, with or without additional Myc-USP39. Consistent with expectation, immunoprecipitation of HA-Ub followed by immunoblotting for Flag-STING showed that USP39 was markedly reduced ubiquitin chains conjugated STING (New Figure S2I). We have added the new results and new indicated cite in our revised manuscript (please see Page 12, Line 318-326, new cite 42).

In Fig. 7E, indeed, it seems that the ubiquitin smears all have the same migration pattern despite the mutation of specific ubiquitination sites on STING. However, USP39 significantly reduced the intensity of the ubiquitin smears of STING. This results might suggest that USP39 regulates most STING deubiquitination, but there are still some STING whose ubiquitin chains are not regulated, which may be related to the activation and conformation of the STING.

Furthermore, according to your suggestion, we have added a brief discussion of the limitation of this part of the study in our revised manuscript (please see Page 15, Line 418-422). The brief discussion was added in the Discussion section, as shown below.

“Furthermore, although we have performed numerous experiments to demonstrate that USP39 can stabilize STING through deubiquitination, we are currently unable to fully prove that USP39 can only exert its effect by removing the covalently linked ubiquitin chains of STING. Further research is needed to confirm this.”

> 
*> Minor comment: "Extron" instead of "Exon" is still used in Figure S2A,B.*

**Response:** Thank you for pointing out this typo. We apologize for the oversight and have revised "Extron" to "Exon" in Figures S2A and S2B accordingly.
