# Supplementary material for: USP39 promotes antiviral defense through post-transcriptional control of RIG-I and stabilization of STING
Source: PLoS Biol. 2026 May 11;24(5):e3003796. doi: 10.1371/journal.pbio.3003796 (PMC13178990; doi:10.1371/journal.pbio.3003796)
Supplement: Attachment — Submitted filename: P to P.docx [file pbio.3003796.s009.docx]

**Answer to the policy-related requests**

*1) We routinely suggest changes to titles to ensure maximum accessibility for a broad, non-specialist readership, and to ensure they reflect the contents of the paper. In this case, we would suggest a minor edit to the title, as follows. Please ensure you change both the manuscript file and the online submission system, as they need to match for final acceptance:*

*> "USP39 promotes antiviral defense through post-transcriptional control of RIG-I and stabilization of STING"*
**Response:** Thanks for your suggestion. We have revised the titles as recommended. (please see the Line 1-2).

*2) The Ethics statement needs to be a separate, independent (and the first) subheading in the Material & Methods section. It must include the full name of the IACUC/ethics committee that reviewed and approved the animal care and use, as well as the protocol/permit/project license number. https://journals.plos.org/plosbiology/s/ethical-publishing-practice*

**Response：**According to your comments, we have added the *Ethics statement* with a separate, independent (and the first) subheading in the Material & Methods section. The Ethics statement have included the full name of the IACUC/ethics committee that reviewed and approved the animal care and use, as well as the project license number in the revised manuscript (please see page 18, line 467-473).

*3) You may be aware of the PLOS Data Policy, which requires that all data be made available without restriction: http://journals.plos.org/plosbiology/s/data-availability. For more information, please also see this editorial: http://dx.doi.org/10.1371/journal.pbio.1001797*

*> Please supply the numerical values either in the a supplementary file or as a permanent DOI’d deposition for the following figures:*

*> Figure 1A-N, 2A-J, 3A-HJ-Q, 4G, 5A-N, 6AF, 7AB, S2B-GI, S3C-FH*

*> NOTE: the numerical data provided should include all replicates AND the way in which the plotted mean and errors were derived (it should not present only the mean/average values).*

**Response：**Thank you for your suggestion. We have provided the numerical data from all replicates in the S1 Data. The statistical methods used to calculate means, error bars, and significance levels are stated in the Materials and Methods section.

*4) Please cite the location of the data clearly in all relevant main and supplementary Figure legends, e.g. “The data underlying this Figure can be found in S1 Data” or “The data underlying this Figure can be found in https://doi.org/10.5281/zenodo.XXXXX”*

**Response：**Thanks for your comments. According your guidance, we have clearly cited the location of the data in all relevant main and supplementary figure legends. The corresponding data sources are specified in the revised manuscript (please see the figure legends for details).

*5) Please ensure that you are using best practice for statistical reporting and data presentation. These are our guidelines https://journals.plos.org/plosbiology/s/best-practices-in-research-reporting#loc-statistical-reporting and a useful resource on data presentation https://journals.plos.org/plosbiology/article?id=10.1371/journal.pbio.1002128*

*> If you are reporting experiments where n ≤ 5, please plot each individual data point.*

**Response:** Thanks for your suggestion. We have carefully reviewed the provided guidelines on best practices for statistical reporting and data presentation and the relevant resource on data visualization. Our statistical reporting and data presentation are following standard scientific best practices, with detailed descriptions provided in the Statistical Analysis subsection under Materials and Methods. In addition, we have plotted each individual data point for all experiments with n ≤ 5 in the revised figures.

*6) Thank you for providing the original, uncropped and minimally adjusted images supporting all blot and gel results reported. However, some of the gels still look cropped, as en example see 4B(MAVS) C(HA-USP39) H(STING). If they are uncropped, then it is ok but please clarify. Please carefully read our guidelines for how to prepare and upload this data: https://journals.plos.org/plosbiology/s/figures#loc-blot-and-gel-reporting-requirements*

**Response:** Thanks for your comment. We have re-uploaded the adjusted images for Figure 4B (MAVS), 4C (HA-USP39), and 4H (STING) with modified display size to clearly show they are uncropped. All other Western blot images presented in the manuscript remain unchanged and are original, uncropped, and minimally processed.

*7) Supplementary files (e.g., excel). Please ensure that all data files are uploaded as 'Supporting Information' and are invariably referred to (in the manuscript, figure legends, and the Description field when uploading your files) using the following format verbatim: S1 Data, S2 Data, etc. Multiple panels of a single or even several figures can be included as multiple sheets in one excel file that is saved using exactly the following convention: S1_Data.xlsx (using an underscore).*

**Response:** Thank you for your guidance. We have organized the raw data into Excel spreadsheets and PDF files, which have been named S1_Data.xlsx and S1_Raw Images.pdf respectively and uploaded as Supporting Information in strict accordance with the required format.

*8) Please add a scale bar in all microscopy pictures*

**Response:** According to your suggestion, we have added a scale bar to all microscopy images, and the size of the scale bar is clearly in the corresponding figure legends.

*9 Please ensure that your Data Statement in the submission system accurately describes where your data can be found and is in final format, as it will be published as written there*

**Response:** Thank you for your comment. We have carefully reviewed and finalized the Data Statement in the submission system, which accurately states the availability and storage location of all data.

*10) Per journal policy, if you have generated any custom code during the course of this investigation, please make it available without restrictions. Please ensure that the code is sufficiently well documented and reusable, and that your Data Statement in the Editorial Manager submission system accurately describes where your code can be found. More information on our Code Policy, what and how to share can be found here: https://journals.plos.org/plosbiology/s/code-availability*

**Response:** Thank you for your comment. We did not produce or use any custom code in this study.

**Answer to the technical requests**

*1) Your current Financial Disclosure states, “This work was supported by grants from the National Natural Science Foundation of China (https://www.nsfc.gov.cn/)(82371788 to WL.C), the Natural Science Foundation of Guangdong Province (http://gdstc.gd.gov.cn)(2024A1515013162 to WL.C; 2024A1515012851 to XB.Z; 2023A1515012402 to YX), the Shenzhen Science and Technology Innovation Program (http://stic.sz.gov.cn/)(KQTD20210811090219022 to WL.C), Medical Science and Technology Research Foundation of Guangdong Province (https://wsjkw.gd.gov.cn)(A2023209 to XB.Z) and Program for Youzuzhikeyan of Shenzhen University (https://www.szu.edu.cn)(to WL.C). The funders had no role in study design, data collection and analysis, decision to publish, or preparation of the manuscript.”*

*However, your funding information on the submission form the funder “Program for Youzuzhikeyan of Shenzhen University" is currently missing from the funding information. Please indicate by return email the full and correct funding information for your study and confirm the order in which funding contributions should appear.*

**Response:** Thank you for your comment. We have supplemented the full and accurate funding information for this study and confirmed the presentation order of all funding sources.

*2) We do not publish any copyright or trademark symbols that usually accompany proprietary names, eg (R), (C), or TM (e.g. next to drug or reagent names). Please remove all instances of trademark/copyright symbols throughout the text, including [®] on line no [500].*

**Response:** Thank you for your comment. We have removed all trademark and copyright symbols throughout the text.

*3)* *We have noticed that you have uploaded Supporting Information files (Original data-1 and Original data2), but you have not included a list of legends.*

*Please add a full list of legends for your Supporting Information files after the references list.*

**Response:** Thanks for your suggestion. We have added a full list of legends for the Supporting Information files after the references list (please see page 22, line 607-655).

*4) Please refer to the following article for PLOS Biology's best practice recommendations for data presentation: https://journals.plos.org/plosbiology/article?id=10.1371/journal.pbio.1002128. Guidelines for statistical reporting, if relevant, may be found here: https://journals.plos.org/plosbiology/s/best-practices-in-research-reporting#loc-statistical-reporting.*

**Response:** Thanks for your suggestion, we have carefully reviewed the provided guidelines on best practices for statistical reporting and data presentation and the relevant resource on data visualization. Our statistical reporting and data presentation are following standard scientific best practices, with detailed descriptions provided in the Statistical Analysis subsection under Materials and Methods. In addition, we have plotted each individual data point for all experiments with n ≤ 5 in the revised figures.

***5)*** *Some material included in your submission may be copyrighted. According to PLOS’scopyright policy, authors who use figures or other material (e.g., graphics, clipart, maps) from another author or copyright holder must demonstrate or obtain permission to publish this material under the Creative Commons Attribution 4.0 International (CC BY 4.0) License used by PLOS journals. Please closely review the details of PLOS’s copyright requirements here: PLOS Licenses and Copyright. If you need to request permissions from a copyright holder, you may use PLOS's Copyright Content Permission form.*

*> Please respond directly to this email and provide any known details concerning your material's license terms and permissions required for reuse, even if you have not yet obtained copyright permissions or are unsure of your material's copyright compatibility. Once you have responded and addressed all other outstanding technical requirements, you may resubmit your manuscript within Editorial Manager.*

*> Potential Copyright Issues:*

*> i) [Figure 8]: Please confirm whether you drew the images / clip-art within the figure panels by hand. If you did not draw the images, please provide (a) a link to the source of the images or icons and their license / terms of use; or (b) written permission from the copyright holder to publish the images or icons under our CC BY 4.0 license. Alternatively, you may replace the images with open source alternatives. See these open source resources you may use to replace images / clip-art:*

*> - https://commons.wikimedia.org*

*> - https://openclipart.org/"*

**Response:** Thanks for your comment. The images in Figure 8 were created and drawn by ourselves using PowerPoint, and no third-party copyrighted clip-art or images were involved.
